# Admission hyperglycemia, in-hospital glycemic management, and discharge outcomes in acute ischemic stroke: a UAE comprehensive stroke center cohort

**DOI:** 10.3389/fendo.2026.1887754

**Published:** 2026-07-15

**Authors:** Mohammed Hamad Al Kuwaiti, Virgie Guy Pedo, Adnan Agha

**Affiliations:** 1 Department of Internal Medicine, College of Medicine and Health Sciences, United Arab Emirates University, Al Ain, United Arab Emirates; 2 Department of Neurology, Sheikh Tahnoon Bin Mohammed Medical City (STMC), Al Ain, United Arab Emirates; 3 Clinical Trials Unit, College of Medicine and Health Sciences, United Arab Emirates University, Al Ain, United Arab Emirates

**Keywords:** acute ischemic stroke, diabetes mellitus, hyperglycemia, modified Rankin Scale, National Institutes of Health Stroke Scale, SHINE trial, stress hyperglycemia ratio, United Arab Emirates

## Abstract

**Background:**

Hyperglycemia at presentation is common after acute ischemic stroke and is associated with worse functional outcomes. We examined whether admission hyperglycemia independently predicted unfavorable discharge outcomes, quantified adherence to the SHINE trial-aligned management standards, and tested the stress hyperglycemia ratio (SHR) as an alternative exposure variable.

**Methods:**

This single-center retrospective cohort study included 218 consecutive adults with acute ischemic stroke admitted to the Tawam Stroke Center, Al Ain, United Arab Emirates, between July 1 and December 31, 2023. The primary exposure was an admission capillary glucose level >7.8 mmol/L. Two co-primary outcomes were defined *a priori*: unfavorable discharge modified Rankin Scale (mRS) scores of 3–6 and in-hospital death. Glycemic management adherence was analyzed as a parallel process measure across 13 q6-h time points. Multivariable logistic regression was adjusted for the pre-specified nine-covariate set (age, sex, NIHSS severity, and six clinical comorbidities). Bonferroni correction was applied across the two co-primary outcomes (*α* = 0.025).

**Results:**

Admission hyperglycemia (51.7%) was associated with unfavorable mRS (adjusted OR 3.43, 95% CI 1.33–8.87, *p =* 0.011; surviving Bonferroni correction). Mantel–Haenszel pooled OR across NIHSS strata: 3.47 (p<0.001). Inappropriate management occurred in 85.8% of hyperglycemic versus 11.1% of euglycemic patients (adjusted OR 80.59, *p* < 0.001). Routine HbA1c testing identified occult diabetes in 18.4% of patients without prior diagnosis. No severe hypoglycemia occurred.

**Conclusions:**

Admission hyperglycemia tripled the adjusted odds of unfavorable discharge outcome and identified patients receiving non-adherent management. Structured admission orders, q6h monitoring and routine HbA1c testing are immediate, low-cost quality improvement targets for Gulf stroke services.

## Graphical Abstract

**Figure:**
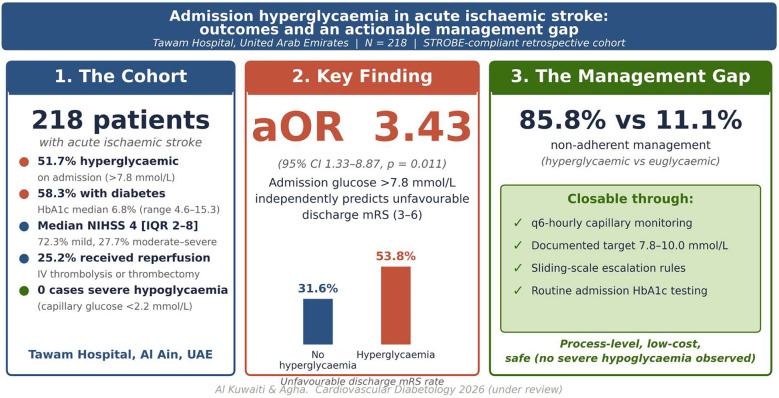
Three-panel visual summary showing (a) the Tawam Stroke Center cohort (218 consecutive acute ischemic stroke admissions, July to December 2023), (b) the principal finding (admission hyperglycemia >7.8 mmol/L was associated with 3.4-fold higher adjusted odds of unfavorable discharge mRS and 86 per cent inappropriate in-hospital glycemic management vs 11 per cent in euglycemic patients), and (c) the implementation message (structured admission orders, q6-hourly glucose monitoring, and routine HbA1c testing as low-cost quality improvement targets for Gulf stroke services). mRS, modified Rankin Scale.

## Background

Hyperglycemia at hospital presentation is documented in approximately 40%–60% of patients with acute ischemic stroke, occurring in those with and without known diabetes (1, 2). Across multiple cohorts and meta-analyses, admission hyperglycemia is associated with larger infarct volumes, hemorrhagic transformation, worse functional outcomes at 90 days, and higher mortality (1, 3, 4). The biological mechanisms include impaired collateral flow, blood–brain barrier disruption, oxidative injury, and conversion of salvageable penumbra to infarction (3, 5). The proportional association with outcomes appears steeper in patients without diabetes, raising the concept of stress hyperglycemia as a distinct prognostic phenomenon (6, 7). The stress hyperglycemia ratio (SHR), defined by Roberts et al. in 2015 as admission glucose divided by the estimated average glucose derived from HbA1c, has emerged as a more biologically informative metric than absolute glucose because it controls for chronic glycemic state (8).

Despite consistent observational evidence, the clinical implications of glycemic control remain unclear. The Stroke Hyperglycemia Insulin Network Effort (SHINE) trial randomized 1,151 hyperglycemic stroke patients to receive intensive intravenous insulin (target 4.4 to 7.2 mmol/L) versus standard subcutaneous insulin (target up to 9.9 mmol/L) for up to 72 h and demonstrated no benefit on the 90-day functional outcome with intensive control, but with a significantly higher rate of severe hypoglycemia in the intensive arm (9). The 2019 American Heart Association/American Stroke Association acute ischemic stroke guideline subsequently recommended treating hyperglycemia to a target of 7.8–10.0 mmol/L using subcutaneous insulin while avoiding hypoglycemia (10). The 2021 American Stroke Association secondary prevention guidelines reinforced the importance of glycemic control for long-term stroke outcomes (11). A 2020 meta-analysis of 12 randomized trials including 2,734 patients confirmed that intensive intravenous insulin protocols do not improve functional outcomes and approximately quintuple the odds of severe hypoglycemia compared with standard subcutaneous insulin regimens (12).

The translation of these recommendations into routine stroke unit practice has not been systematically audited in the Arabian Gulf, where the prevalence of diabetes among adults exceeds 17% in several countries and reaches approximately 19% in the United Arab Emirates, the highest reported in the region (13, 14). The incidence of stroke in the UAE is the highest among the Gulf Cooperation Council states (106.01 per 100,000 in 2021) (14); however, the interaction between hyperglycemia and stroke outcomes in this population is unknown. In 2022, the Department of Health Abu Dhabi established a three-tier stroke network, requiring hospitals to be accredited as stroke-ready, primary, or comprehensive centers to standardize care and enhance regional stroke outcomes (15). Regional thrombolysis utilization and outcomes have been characterized through the SITS-MENA registry (16), and a single-center Al Ain Hospital registry has described quality metrics, demographics, and reperfusion utilization (17), establishing a regional foundation for benchmarking stroke. The current body of literature in the UAE does not encompass studies focused on evaluating in-hospital hyperglycemia management, the relationship between admission glucose and discharge functional status, or adherence to SHINE-aligned protocols, leaving this avenue of acute stroke care in the UAE unexplored. The absence of such evidence limits the design of locally relevant quality improvement interventions. To address this issue, we conducted a retrospective cohort analysis of acute ischemic stroke admissions to Tawam Hospital, a certified comprehensive stroke center in Al Ain. Two clinical outcomes were defined *a priori* as co-primary, namely: (i) unfavorable functional status at hospital discharge, defined by an mRS score of 3–6, and (ii) in-hospital mortality. Adherence to SHINE-aligned glycemic management standards was analyzed in parallel as a process-of-care measure rather than a third clinical outcome, and the stress hyperglycemia ratio (SHR) was examined as a supplementary exposure to test whether the relative excursion above chronic glycemia added prognostic information beyond the absolute admission glucose level. Therefore, the objectives were to quantify the independent association of admission hyperglycemia with each co-primary outcome after full multivariable adjustment, to characterize SHINE-protocol adherence and identify the patient subgroups most likely to experience implementation failure, and to test SHR as a refined exposure variable in the subset with paired admission glucose and HbA1c levels.

## Methods

### Study design

This single-center retrospective cohort study was conducted at Tawam Hospital, a comprehensive stroke center in Al Ain, United Arab Emirates, which was internationally certified by the American Heart Association in December 2023. During the study period, Tawam Hospital served as the principal stroke service for the eastern region of the Emirate of Abu Dhabi, with a 24/7 stroke unit (10 beds) and a hyperacute stroke-capable emergency room providing time-sensitive evaluation and therapies, including intravenous thrombolysis and mechanical thrombectomy in line with the 2019 American Heart Association/American Stroke Association guidelines for the early management of acute ischemic stroke (10).

### Participants and time window

**Figure 1:**
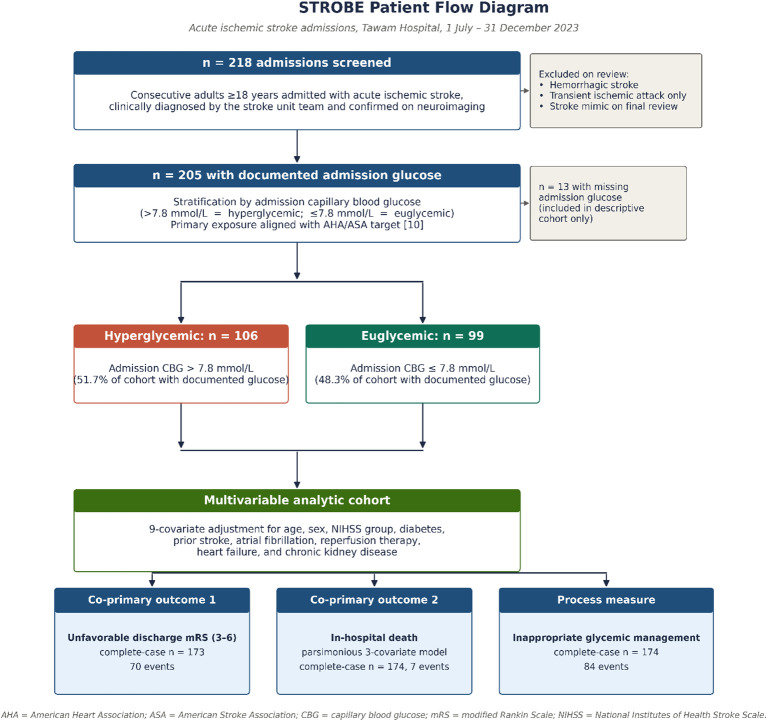
STROBE patient flow diagram. Flow of 218 consecutive acute ischemic stroke admissions (Tawam Hospital, July 1 to December 31, 2023) clinically diagnosed by the stroke unit team and confirmed on neuroimaging, with stratification by admission capillary blood glucose, and into the multivariable analytic cohort. The three pre-specified analytic models (unfavorable discharge mRS, in-hospital death, and inappropriate glycemic management) and their respective complete-case sample sizes and event counts are shown at the bottom. AHA, American Heart Association; ASA, American Stroke Association; CBG, capillary blood glucose; mRS, modified Rankin Scale; NIHSS, National Institutes of Health Stroke Scale.

We included all consecutive adults aged ≥18 years who were admitted to the stroke unit between July 1 and December 31, 2023, with a primary clinical diagnosis of acute ischemic stroke confirmed by neuroimaging. Cases were drawn from the Tawam Hospital institutional stroke registry, which prospectively logs every acute stroke admission as part of routine quality improvement reporting. The registry was the sampling frame for this study and provided a complete enumeration of admissions during the observation period. Patients were excluded only if the principal admission diagnosis was hemorrhagic stroke, transient ischemic attack, or stroke mimic on the final review. The descriptive cohort included 218 patients, of whom 205 had documented admission glucose levels and were included in the primary exposure analysis. This study followed the Strengthening the Reporting of STROBE in Epidemiology guidelines for observational studies (18), and the patient flow diagram is shown in **Figure 1**.

To address potential selection effects, the 13 patients with missing admission glucose werecompared to the analyzed cohort (*n* = 205) across age, sex, stroke severity, length of stay, comorbidities, and clinical outcomes. The two subsets did not differ on any of these characteristics except for a lower diabetes prevalence in the missing-glucose subset (30.8% versus 60.0%; Fisher’s exact *p* = 0.046), most plausibly reflecting that admission glucose was less consistently documented when no known diabetes history prompted the test. Full comparison statistics are reported in **Supplementary Table 1**. The pattern is consistent with missing at random conditional on observed covariates, supporting the use of complete-case analysis for primary models.

### Exposures

The primary exposure was admission capillary blood glucose >7.8 mmol/L (140 mg/dL), measured within 60 min of arrival at the emergency department. This threshold corresponds to the lower limit of the American Heart Association/American Stroke Association-recommended in-hospital glycemic target of 7.8–10.0 mmol/L (10) and is the threshold above which active glycemic management is indicated in contemporary guidelines. As a supplementary exposure aligned with the current literature on stress hyperglycemia, the stress hyperglycemia ratio (SHR) was calculated in patients with both admission glucose and HbA1c documented using the Roberts formula: estimated average glucose (mmol/L) = (1.59 × HbA1c%) − 2.59, with SHR defined as admission glucose divided by estimated average glucose (8). The patients were stratified into SHR tertiles for the analysis. Two alternative absolute glucose thresholds (a split-protocol cutoff of >6.1 mmol/L if known diabetes and >8.3 mmol/L if not, and a higher cutoff of >10 mmol/L) were examined as pre-specified sensitivity analyses; estimates were directionally consistent across both alternatives and were not tabulated.

### Outcomes

Two clinical co-primary outcomes were defined in this study. The first was unfavorable functional status at hospital discharge, defined *a priori* as a modified Rankin Scale (mRS) score of 3–6 (moderate disability or worse, including death) versus 0–2 (functional independence or slight disability) (19). The second was in-hospital death, captured by discharge disposition coded as deceased.

Glycemic management adherence was analyzed as a parallel implementation outcome rather than a third clinical co-primary outcome on the rationale that it is a process-of-care quality measure rather than a patient-level health outcome. It was derived algorithmically from every 6 h (q6h) of capillary blood glucose record and matched sliding-scale insulin documentation across 13 pre-specified time points from presentation to 72 h. A patient was classified as receiving inappropriate management if any of the following occurred: (i) any documented capillary glucose of ≥10 mmol/L without sliding-scale insulin administered at the same timepoint, (ii) capillary glucose of ≥10 mmol/L with sliding-scale insulin administered, but the next reading remained at or above 10 mmol/L without dose escalation, or (iii) lowest capillary glucose <2.2 mmol/L without documented corrective treatment. Patients with no glucose readings ≥10 mmol/L and no severe hypoglycemia were classified as appropriate by default.

Unfavorable 90-day mRS was pre-specified as an exploratory secondary outcome but was not analyzed as co-primary because of substantial loss to follow-up; the results in the available subset are reported separately.

### Covariates

The full adjustment set was specified before the analysis: age (years, continuous), sex (female versus male), stroke severity by the National Institutes of Health Stroke Scale (NIHSS) (20), with NIHSS groups (mild 0–7, moderate 8–14, severe ≥15; aligned with the SHINE trial three-tier stratification (9), modified to include NIHSS 0–2 within the mild stratum so that very mild but objectively documented strokes contributed to the multivariable analysis), known diabetes mellitus, prior stroke or transient ischemic attack, atrial fibrillation, heart failure, chronic kidney disease, and receiving reperfusion therapy (intravenous tissue plasminogen activator, mechanical thrombectomy, or both versus none). Where NIHSS was undocumented, the patient contributed to univariate descriptive analyses but not to the fully adjusted multivariable model; the proportion missing is reported transparently in the “Results” section.

### Data extraction and quality control

Data were extracted from the electronic medical records by two trained abstractors using a piloted case report form, with a 10% double-extraction sample to assess agreement. Capillary glucose values, sliding-scale insulin records, and mRS scores were verified using the primary chart entries. Two extreme but plausible admission glucose values (41.5 and 31.8 mmol/L) were retained after confirming the HbA1c levels and known diabetes status. Severe hypoglycemia (lowest glucose <2.2 mmol/L) was independently assessed across all glucose records as a safety endpoint.

Continuous variables were assessed for normality using Shapiro–Wilk test and reported as mean with standard deviation if normally distributed or median with interquartile range if not. Categorical variables are expressed as counts and column percentages. Group comparisons were performed using Mann–Whitney *U*-test for continuous variables (given the non-normal distributions of glucose and HbA1c) and chi-square test or Fisher’s exact test for categorical variables, with Fisher’s exact test applied when any expected cell frequency was below 5. Crude associations between admission hyperglycemia and each outcome were estimated as cross-product odds ratios (ORs) with Wald 95% confidence intervals (CIs). Fisher’s exact two-sided *p*-values are reported as a sensitivity check given sparse cells for the mortality outcome. The sample size was determined by the cohort of consecutive eligible admissions within the pre-specified 6-month observation window; no *a priori* power calculation was undertaken. The *post-hoc* power for the two clinical co-primary outcomes is reported in the “Results” section.

### Multivariable model construction

Adjusted associations were estimated using binary logistic regression analysis. The full adjustment set was specified before the analysis on clinical grounds rather than data-driven selection: age (continuous), sex (female vs. male), NIHSS group (mild 0–7 reference, moderate 8–14, severe ≥15), known diabetes mellitus, prior stroke or transient ischemic attack, atrial fibrillation, heart failure, chronic kidney disease, and receipt of any acute reperfusion therapy. One model was fitted for each of the outcomes. The events-per-variable rule was respected for the primary discharge mRS outcome (70 events, nine covariates, 7.8 events per variable) and for the inappropriate management process measure (84 events, five covariates, and 16.8 events per variable). For the in-hospital death outcome, given event sparsity (14 deaths), the adjustment set was reduced to admission hyperglycemia, age, and NIHSS group only.

Covariates were selected on a pre-specified conceptual model in which admission hyperglycemia could affect outcomes through (i) direct cytotoxic effects on brain tissue (mediated by stroke severity captured by NIHSS group), (ii) shared pathophysiology with cardiometabolic comorbidities (diabetes mellitus, atrial fibrillation, heart failure, chronic kidney disease, prior stroke), and (iii) intersection with acute reperfusion treatment. Age and sex were included as standard confounders. NIHSS group was treated as a baseline severity covariate rather than a downstream mediator on the assumption that it captures pre-existing severity at presentation rather than a consequence of the exposure.

### Model diagnostics and assumption checks

Three diagnostics were applied to the primary discharge mRS multivariate model. First, multicollinearity was assessed by calculating the variance inflation factors (VIFs) for each covariate using a linear regression proxy, and values below 5 were considered acceptable per conventional thresholds (21), with values above 10 considered concerning. Second, model calibration was assessed using Hosmer–Lemeshow goodness-of-fit test, with a non-significant *p*-value indicating that the observed and model-predicted event probabilities did not differ across deciles of risk. Third, model discrimination was assessed by the receiver operating characteristic (ROC) curve generated from the model’s predicted probabilities, with the area under the curve (AUC) and 95% confidence interval reported; AUC ≥0.70 was considered acceptable, ≥0.80 was excellent, and ≥0.90 was outstanding (22).

### Confounding factors included stroke severity

Stroke severity is the strongest known prognostic determinant of acute ischemic stroke and could confound the hyperglycemia–outcome association; therefore, two complementary approaches were applied in this study. First, the NIHSS group was included as a categorical covariate in each multivariable model. Second, Mantel–Haenszel stratified analysis was performed across the three NIHSS strata to compute a pooled common odds ratio independent of the regression model, using Breslow–Day test to assess the homogeneity of the stratum-specific estimates. Concordance between the regression-adjusted and Mantel–Haenszel-pooled estimates would indicate that the principal finding was not driven by residual severity confounding within the regression specifications.

### Sensitivity analyses

As a sensitivity analysis of covariate specification, the primary discharge mRS multivariable model was re-fitted with NIHSS as a continuous covariate (per one-point increase) in place of the three-level NIHSS group categorical variable, and the change in Akaike Information Criterion was reported to compare the model fit. As a sensitivity analysis of the exposure definition, the primary model was re-fitted using two alternative admission glucose thresholds: a split-protocol cutoff (above 6.1 mmol/L if diabetes is known, whereas above 8.3 mmol/L if not) and an exploratory upper threshold (above 10 mmol/L). A complete case sensitivity analysis was performed to confirm that the missing data did not materially alter the primary estimates.

To address near-complete separation in the inappropriate-management model and small-sample biasin the mortality model, a Firth-penalized logistic regression sensitivity analysis was pre-specifiedfor all three multivariable models. Firth’s penalty modifies the likelihood by a Jeffreys prior term that produces finite estimates under separation and reduces small-sample bias (33, 34). Firth regression was implemented in Python 3 using the scipy and statsmodels packages; the full Firth-penalized coefficient tables for all three primary models are reported in **Supplementary Table 2**. The unpenalized maximum-likelihood estimates remain the primary report for consistency with the pre-specified analysis plan; the Firth estimates are interpreted as a robustness check.

### Stress hyperglycemia ratio analysis

The SHR was analyzed in two parallel specifications: as a categorical variable (tertile 1 reference, tertile 2, and tertile 3) and as a continuous variable (per 0.1-unit increase). The same nine-covariate adjustment set was used, substituting SHR for binary admission hyperglycemia exposure. The linear trend across the SHR tertiles was tested by entering the tertile ranks as ordinal terms.

### Significance thresholds and *post-hoc* power

A two-sided alpha was set at 0.05 throughout, and a Bonferroni-corrected alpha of 0.025 was applied across the two clinical co-primary outcomes for the principal multivariable estimates. Glycemic management adherence, analyzed as a parallel process measure, was reported without a joint Bonferroni correction. The *post-hoc* statistical power for each co-primary outcome was calculated using a two-proportion *z*-test based on the observed event rates and group sizes; this calculation is reported in the “Strengths and limitations” section. All analyses, including multivariable models, sensitivity analyses, and Mantel–Haenszel pooling, were performed using IBM SPSS Statistics version 31 (IBM Corp., Armonk, NY, USA).

### Use of generative artificial intelligence

Generative AI tools (Anthropic Claude, Google Gemini, and Paperpal AI) were used for language editing and manuscript formatting only under the direct supervision of the corresponding author; the full disclosure statement, including scope and accountability, is provided in the declarations.

### Ethical approval

The study was approved by Tawam Hospital Research Ethics Committee on February 21, 2024 (ethics committee approval number MF2058-2024-1050). Because of the retrospective chart review design and fully de-identified analytic dataset, the requirement for individual informed consent was waived in accordance with the institutional policy and the Declaration of Helsinki.

## Results

The descriptive cohort comprised 218 adults with confirmed acute ischemic stroke, of whom 205 had documented admission glucose measurements. The baseline demographic and clinical characteristics according to admission glycemic status are presented in **Table 1**. The hyperglycemic group had a higher diabetes prevalence and HbA1c level; however, the NIHSS scores at presentation did not differ between the groups.

**Table 1:** Baseline characteristics, treatment, glycemic profile, and crude outcomes by admission glycemic status.

Variable	Hyperglycemic (*n* = 106)	Euglycemic (*n* = 99)	*p*-value
Demographics and past medical history			
Age, years, mean ± SD	61.0 ± 14.6	55.5 ± 15.0	0.005
Female sex, *n* (%)	23 (21.7)	25 (25.3)	0.660
Ethnicity, *n* (%)			
UAE national	30 (28.3)	16 (16.2)	0.052
Arab non-UAE	26 (24.5)	30 (30.3)	
Asian	49 (46.2)	53 (53.5)	
Other	1 (0.9)	0 (0.0)	
BMI, kg/m², mean ± SD	27.7 ± 5.2	26.9 ± 5.7	0.213
Diabetes mellitus, *n* (%)	88 (83.0)	36 (36.4)	<0.001
Hypertension, *n* (%)	85 (80.2)	60 (60.6)	0.002
Hyperlipidemia, *n* (%)	59 (55.7)	37 (37.4)	0.013
Smoking ever, *n* (%)	23 (21.7)	26 (26.3)	0.534
Prior stroke or TIA, *n* (%)	29 (27.4)	12 (12.1)	0.011
Atrial fibrillation, *n* (%)	15 (14.2)	3 (3.0)	0.008
Heart failure, *n* (%)	10 (9.4)	1 (1.0)	0.014
Chronic kidney disease, *n* (%)	16 (15.1)	3 (3.0)	0.005
Stroke characteristics, time to presentation, treatment, and crude outcomes			
NIHSS at presentation, median [IQR]	5 [3–9]	4 [2–7]	0.094
NIHSS group, *n* (%)			
Mild (0–7)	62 (58.5)	62 (62.6)	0.643
Moderate (8–14)	12 (11.3)	11 (11.1)	
Severe (≥15)	15 (14.2)	11 (11.1)	
NIHSS undocumented	17 (16.0)	15 (15.2)	
Time to ED, ≤24 h, *n* (%)	73 (68.9)	76 (76.8)	0.297
Reperfusion therapy, *n* (%)	28 (26.4)	26 (26.3)	1.000
IV thrombolysis only	12 (11.3)	11 (11.1)	
Mechanical thrombectomy only	9 (8.5)	10 (10.1)	
Combined IV + MT	7 (6.6)	5 (5.1)	
Length of stay, days, median [IQR]	6 [4–11]	4 [3–8]	<0.001
Unfavorable discharge mRS (3–6), *n* (%)	57 (53.8)	31 (31.6)	0.002
In-hospital death, *n* (%)	11 (10.4)	3 (3.0)	0.051
Inappropriate management, *n* (%)	91 (85.8)	11 (11.1)	<0.001
Glucose, HbA1c, stress hyperglycemia ratio, and in-hospital glycemic burden			
Admission glucose, mmol/L, median [IQR]	11.7 [9.4–15.4]	6.0 [5.4–6.7]	<0.001
HbA1c documented, *n* (%)	91 (85.8)	87 (87.9)	0.831
HbA1c, %, median [IQR]	8.6 [7.0–10.8]	5.9 [5.7–6.3]	<0.001
Stress hyperglycemia ratio, median [IQR]	1.10 [0.94–1.37]	0.86 [0.76–0.96]	<0.001
HbA1c ≥6.5% in no-known-DM (occult DM), *n* (%)	9/15 (60.0)	5/53 (9.4)	<0.001
Highest in-hospital glucose, mmol/L, median [IQR]	15.5 [11.4–18.8]	7.4 [6.4–8.9]	<0.001
Lowest in-hospital glucose <2.2 mmol/L, *n* (%)	0 (0.0)	0 (0.0)	—

Hyperglycemia is defined as admission capillary blood glucose >7.8 mmol/L. Continuous variables were compared using Mann–Whitney *U*-test, whereas categorical variables were compared using chi-square or Fisher’s exact test as appropriate. Stress hyperglycemia ratio was calculated using the Roberts formula: estimated average glucose (mmol/L) = (1.59 × HbA1c%) – 2.59, with SHR = admission glucose ÷ estimated average glucose. Occult diabetes is defined as HbA1c ≥6.5% in patients without prior diabetes diagnosis.

BMI, body mass index; ED, emergency department; HbA1c, glycated hemoglobin; IQR, interquartile range; IV, intravenous; mRS, modified Rankin Scale; MT, mechanical thrombectomy; NIHSS, National Institutes of Health Stroke Scale; SHR, stress hyperglycemia ratio; TIA, transient ischemic attack; UAE, United Arab Emirates.

### Co-primary clinical outcome 1: unfavorable discharge mRS

Discharge mRS was documented in 217 of 218 patients (99.5%); among those with documented mRS, 95 (43.8%) had an unfavorable outcome (mRS 3–6). Among the 204 of 218 (93.6%) patients with both admission glucose and discharge mRS available, unfavorable outcomes occurred in 57 of 106 hyperglycemic patients (53.8%) and in 31 of 98 euglycemic patients (31.6%)—cross-product OR 2.51 (95% CI 1.42–4.45), Fisher’s exact *p =* 0.002 (**Table 2**). The 13 patients with missing admission glucose levels were retained in the descriptive cohort but excluded from this stratified comparison.

**Table 2:** Crude and adjusted associations between admission hyperglycemia and the two clinical co-primary outcomes.

Outcome	Hyper­glycemic events (%)	Euglycemic events (%)	Crude OR (95% CI)	Adjusted OR (95% CI)	*p* (adjusted)
Co-primary 1: unfavorable discharge mRS	57/106 (53.8)	31/98 (31.6)	2.51 (1.42–4.45)	3.43 (1.33–8.87)^a^	0.011
Co-primary 2: in-hospital death	11/106 (10.4)	3/99 (3.0)	3.71 (1.00–13.70)	5.61 (0.65–48.43)	0.117
Process measure: inappropriate management	91/106 (85.8)	11/99 (11.1)	48.5 (21.1–111.5)	80.59 (20.21–321.4)	<0.001

The cross-product OR was calculated using Wald 95% CI. Fisher’s exact two-sided *p*-values were 0.002, 0.051, and <0.001, respectively. The unfavorable discharge mRS adjusted model includes age, sex, NIHSS group (mild 0–7 reference), diabetes, prior stroke, atrial fibrillation, reperfusion therapy, heart failure, and chronic kidney disease (complete-case *n* = 173, 70 events). The in-hospital death adjusted model is a parsimonious three-covariate model (admission hyperglycemia, age, and NIHSS group) given event sparsity (14 deaths in cohort; seven events in the complete-case sample of *n* = 174). The inappropriate management adjusted model includes age, sex, NIHSS group, and diabetes (complete-case *n =* 174, 84 events). The full coefficient tables for all three models are provided in **Table 3**, sections A, B, and C, respectively.

CI, confidence interval; mRS, modified Rankin Scale; OR, odds ratio.

^a^ Significant at the Bonferroni-corrected threshold (*α* = 0.025) applied across the two clinical co-primary outcomes.

In the primary multivariable logistic regression model (complete-case *n =* 173, 70 unfavorable events; covariates listed in “Methods”), admission hyperglycemia was independently associated with unfavorable discharge mRS, with an adjusted OR of 3.43 (95% CI 1.33–8.87, *p =* 0.011), surviving the Bonferroni-corrected threshold of α = 0.025 (**Table 3**). The only other independent associated factors were NIHSS severity strata: adjusted OR 4.95 (95% CI 1.64–14.98, *p* = 0.005) for moderate stroke (NIHSS 8–14) and adjusted OR 20.91 (95% CI 4.90–89.17, *p* < 0.001) for severe stroke (NIHSS ≥ 15), both relative to mild stroke (NIHSS 0–7) as the reference. Age, sex, diabetes, prior stroke, atrial fibrillation, reperfusion therapy, heart failure, and chronic kidney disease were not independent associated factors after adjustment (**Table 3**). Pre-specified sensitivity analyses using two alternative admission glucose thresholds, asplit-protocol cutoff (>6.1 mmol/L if known diabetes, whereas >8.3 mmol/L if not) and >10mmol/L, produced directionally consistent estimates (**Supplementary Table 3**). Firth-penalized sensitivity analysis on the same complete-case sample confirmed theassociation (adjusted OR 3.10, 95% confidence interval 1.25–7.71,*p* = 0.015; **Supplementary Table 2**), with consistent point estimates and tighter CIs for the NIHSS coefficients.

**Table 3:** Full coefficient tables from the three pre-specified adjusted multivariable logistic regression models.

Covariate	Adjusted OR	95% CI	*p*-value
A. Unfavorable discharge mRS (3–6)—co-primary outcome 1; complete-case *n =* 173, 70 events			
Admission hyperglycemia (>7.8 mmol/L)	3.43	1.33–8.87	0.011
Age (per year)	1.02	0.99–1.05	0.106
Female sex	1.27	0.51–3.16	0.601
NIHSS group: moderate (8–14)	4.95	1.64–14.98	0.005
NIHSS group: severe (≥15)	20.91	4.90–89.17	<0.001
Diabetes mellitus	1.26	0.45–3.54	0.655
Prior stroke/TIA	1.38	0.53–3.58	0.505
Atrial fibrillation	0.45	0.08–2.63	0.373
Reperfusion therapy	1.47	0.56–3.85	0.433
Heart failure	1.42	0.29–6.89	0.667
Chronic kidney disease	0.74	0.19–2.91	0.663
B. In-hospital death—co-primary outcome 2; parsimonious three-covariate model; complete-case *n =* 174, 7 events			
Admission hyperglycemia (>7.8 mmol/L)	5.61	0.65–48.43	0.117
Age (per year)	1.03	0.98–1.09	0.256
NIHSS group: moderate (8–14)	3.71	0.56–24.63	0.175
NIHSS group: severe (≥15)	2.49	0.36–17.15	0.355
C. Inappropriate glycemic management—process measure; complete-case *n =* 174, 84 events			
Admission hyperglycemia (>7.8 mmol/L)	80.59	20.21–321.38	<0.001
Diabetes mellitus	45.38	8.79–234.40	<0.001
NIHSS group: moderate (8–14)	3.90	0.56–27.25	0.170
NIHSS group: severe (≥15)	2.11	0.47–9.38	0.328
Age (per year)	1.00	0.96–1.04	0.980
Female sex	0.16	0.03–0.77	0.023

Reference categories throughout: NIHSS group mild (0–7); male sex; all other covariates absent. Section A: Hosmer–Lemeshow chi-square = 3.82, df = 8, *p =* 0.873; all variance inflation factors below 5. Section B: parsimonious adjustment (admission hyperglycemia, age, and NIHSS group only) given event sparsity (14 deaths in the descriptive cohort; seven events in the complete-case multivariable sample). Section C: HbA1c was omitted from this model to preserve the sample size; its predictive content overlaps with diabetes status and admission glucose, both of which are retained. Wide confidence intervals for the hyperglycemia and diabetes effects in section C reflect near-complete separation.

CI, confidence interval; mRS, modified Rankin Scale; NIHSS, National Institutes of Health Stroke Scale; OR, odds ratio; TIA, transient ischemic attack.

The Hosmer–Lemeshow goodness-of-fit test for the primary multivariable model showedexcellent calibration (chi-square = 3.82, df = 8, *p =* 0.873), and the receiveroperating characteristic curve for the same primary model demonstrated excellent discrimination (area under the curve 0.814, 95% CI 0.746–0.879; **Supplementary Figure 1**). All variance inflation factors were below 5, indicating no multicollinearity.Mantel–Haenszel stratification by NIHSS group confirmed the hyperglycemia–outcomeassociation across all three severity strata, namely: mild 0–7 (*n =* 124), stratum-specific OR 2.55 (95% CI 1.11–5.87), 22 of 62 hyperglycemic patients vs. 11 of 62 euglycemic patients with unfavorable outcome; moderate 8–14 (*n =* 23), OR 29.33 (95% CI 2.56–336.4), 11 of 12 vs. three of 11, with the wide confidence interval reflecting near-complete outcome separation in the hyperglycemic group rather than residual confounding; and severe ≥15 (*n =* 26), OR 3.11 (95% CI 0.24–39.54), 14 of 15 vs. nine of 11. The pooled Mantel–Haenszel OR was 3.47 (95% CI 1.48–8.10, Mantel–Haenszel chi-square = 12.01, *p* < 0.001), demonstrating that the association was not explained by differences in stroke severity (**Supplementary Figure 2**).

### Co-primary clinical outcome 2: in-hospital death

A total of 14 patients (6.4% of the descriptive cohort) died during index hospitalization; 11 of these (78.6%) had admission hyperglycemia. Among the 205 patients with documented admission glucose, the crude prevalence of in-hospital death was 10.4% in the hyperglycemic group versus 3.0% in the euglycemic group (cross-product OR 3.71, 95% confidence CI 1.00–13.70, Fisher’s exact *p* = 0.051; **Table 2**). Because the event count was too small to support the full nine-covariate adjustment under the events-per-variable rule (10 events per covariate maximum), a parsimonious adjusted model was fitted with admission hyperglycemia, age, and NIHSS group only. The full set of coefficients is listed in **Table 3**. Admission hyperglycemia was not an independent associated factor of in-hospital death afteradjustment (*p* = 0.117), nor were age(*p* = 0.256) and NIHSS group (moderate, *p* = 0.175; severe, *p* = 0.355). In the Firth-penalized sensitivity analysis on the same parsimonious sample (*n* = 174, seven events), the adjusted OR for admission hyperglycemia was 3.76 (95% confidence interval 0.70–20.25, *p* = 0.123), with the upper CI tightened by approximately 58% compared to the unpenalized estimate (**Supplementary Table 2**). Mantel–Haenszel severity-stratified pooling could not be performed for this outcome because two of the three NIHSS strata contained zero deaths in the euglycemic group, rendering stratum-specific estimates inestimable without continuity correction. The *post-hoc* statistical power for the observed mortality effect was 59.1% at *α* = 0.05 and 48.4% at the Bonferroni-corrected *α* = 0.025, indicating that the cohort was underpowered to detect this effect at conventional significance thresholds; the implications for interpretation are addressed in the “Strengths and limitations” section.

### Process measure: inappropriate glycemic management

**Figure 2:**
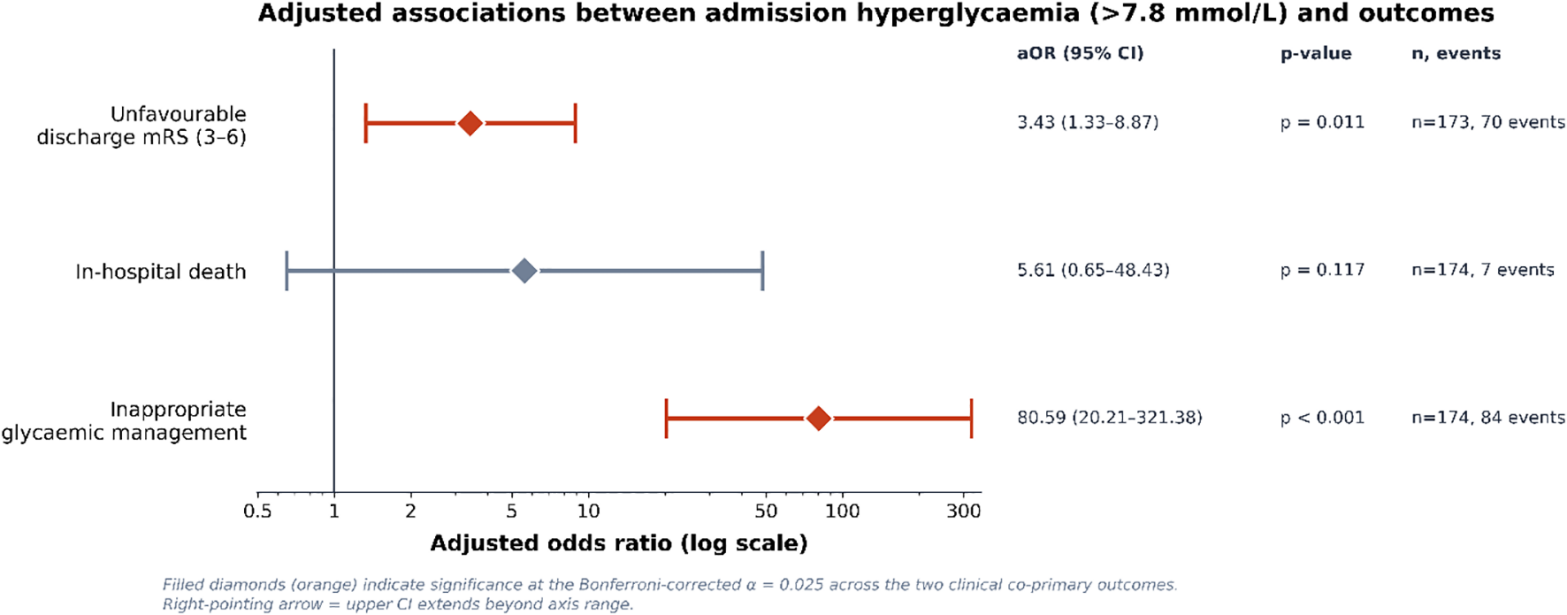
Forest plot of adjusted odds ratios for admission hyperglycemia on the two clinical co-primary outcomes. The figure shows a forest plot of adjusted odds ratios for admission hyperglycemia (>7.8 mmol/L) on the two clinical co-primary outcomes and the parallel process measure. Adjusted ORs and 95% confidence intervals are displayed on a logarithmic axis. Filled diamonds (orange) indicate significance at the Bonferroni-corrected *α* = 0.025 across the two clinical co-primary outcomes. The right-pointing arrow indicates that the upper confidence limit extends beyond the displayed axis range. Full coefficient tables are presented in **Tables 3**-**5**. CI, confidence interval; mRS, modified Rankin Scale; OR, odds ratio.

Using the algorithm-derived metric, 104 of 218 patients (47.7%) had at least one episode of inappropriate glycemic management during admission. The prevalence was concentrated in the hyperglycemic group: 91 of 106 hyperglycemic patients (85.8%) versus 11 of 99 euglycemic patients (11.1%); the remaining two inappropriate-management episodes occurred in patients with no documented admission glucose, who were therefore excluded from the stratified comparison in **Table 1** but counted in the overall denominator (*n* = 218). The most commonprotocol failure was a documented capillary glucose level at or above 10 mmol/L without simultaneoussliding-scale insulin documentation, accounting for 247 of 260 inappropriate-management episodes (95.0%); the remaining 13 episodes (5.0%) were readings of glucose below 4 mmol/L without a documented hypoglycemia response (**Supplementary Table 4**). Severe hypoglycemia, defined as the lowest in-hospital glucose level below 2.2 mmol/L, was not observed in any patient, providing a clear safety signal for the predominantly subcutaneous insulin pathway used in this center. The adjusted odds ratios for the two clinical co-primary outcomes and the inappropriate management process measure are summarized in **Figure 2**.

The full multivariable logistic regression model for inappropriate management (**Table 3**) included admission hyperglycemia, diabetes, NIHSS group, age, and sex; HbA1c was omitted topreserve the sample size, given that its predictive content overlaps with diabetes status andadmission glucose levels. In a complete-case sample of 174 patients with 84 events, admission hyperglycemia was the strongest independent associated factor (adjusted OR 80.59, 95% confidence CI 20.21–321.38, *p* < 0.001), followed by diabetes (adjusted OR 45.38, 95% confidence CI 8.79–234.40, *p* < 0.001). Female sex was associated with lower odds of inappropriate management (adjusted OR 0.16, 95% confidence CI 0.03–0.77, *p =* 0.023). The NIHSS group and age were not independent associated factors after adjustment for confounding factors. The very wide confidence intervals for the hyperglycemia and diabetes coefficients reflect near-complete separation between exposure groups (almost all hyperglycemic patients and almost all patients with diabetes experienced inappropriate management); therefore, the point estimates are best interpreted as evidence of a strong association rather than precise effect size estimates. The univariate associations are listed in **Supplementary Table 5**. Firth-penalized regression on the same complete-case sample (*n =* 174, 84events) shrank the point estimates and CI widths while preserving direction and significance for allthree independent associations: admission hyperglycemia (adjusted OR 53.75, 95% CI 15.66–184.49), diabetes (adjusted OR 31.47, 95% CI 7.16–138.34), and female sex (adjusted OR 0.18, 95% CI 0.04–0.79, *p =* 0.023). The Firth coefficient table is provided in **Supplementary Table 2**.

The pattern of inappropriate management across NIHSS strata and hyperglycemia status is shown in**Supplementary Figure 3**, which demonstrates that protocol failure is concentrated in hyperglycemic patients regardless of stroke severity stratum. The pattern identified a clear quality improvement target: the patients who most need protocolized management (those with established diabetes and elevated presenting glucose) are precisely those most likely to experience protocol deviations.

### Stress hyperglycemia ratio

The stress hyperglycemia ratio was calculated for 166 patients with documented admission glucose and HbA1c levels. The median SHR was 0.95 (interquartile range 0.81–1.17), with values above 1.0 indicating that the admission glucose level exceeded the chronic glycemic baseline predicted from the HbA1c level. The median SHR was higher in the hyperglycemic group (1.10, IQR 0.94–1.37) than in the euglycemic group (0.86, IQR 0.76–0.96; Mann–Whitney *p* < 0.001; **Table 1**), reflecting both absolute glucose elevation and relative excursion above the baseline. Tertile boundaries were SHR < 0.873 (T1, *n* = 56), 0.873–1.059 (T2, *n =* 55), and >1.059 (T3, *n =* 55).

Among the 165 patients with SHR and documented discharge mRS, unfavorable functional outcomesincreased monotonically across the SHR tertiles: 26.8% in T1 (15 of 56), 35.2% in T2 (19 of 54), and56.4% in T3 (31 of 55), with a significant linear trend (unadjusted OR per tertile 1.90, *p*-trend = 0.002; **Supplementary Figure 4**). After adjusting for the same nine covariates as the primary model, the OR for T3 versus T1 was 2.45 (95% CI 0.90–6.66, *p =* 0.078; full coefficients are presented in **Table 4**). When SHR was modeled as a continuous variable, each 0.1-unit increase carried an adjusted OR of 1.08 (95% confidence interval 0.96–1.21, *p =* 0.206). The attenuation of the SHR effect after multivariable adjustment is consistent with the well-documented overlap between SHR and known diabetes status (adjusted OR for diabetes within the same model 3.34, *p =* 0.017) together with NIHSS severity, both of which capture related glycemic and prognostic information that partially accounts for the unique contribution of SHR.

**Table 4:** Multivariable logistic regression for unfavorable discharge using stress hyperglycemia ratio tertiles as the exposure.

Covariate	Adjusted OR	95% CI	*p*-value
SHR tertile 1 (≤0.873)	1.00 (ref)	—	—
SHR tertile 2 (0.873–1.059)	1.32	0.47–3.71	0.595
SHR tertile 3 (>1.059)	2.45	0.90–6.66	0.078
Age (per year)	1.00	0.97–1.04	0.829
Female sex	0.83	0.30–2.29	0.717
NIHSS group: moderate (8–14)	4.07	1.21–13.74	0.024
NIHSS group: severe (≥15)	34.88	5.95–204.44	<0.001
Diabetes mellitus	3.34	1.24–8.95	0.017
Prior stroke/TIA	1.10	0.37–3.28	0.867
Atrial fibrillation	0.43	0.05–3.50	0.434
Reperfusion therapy	1.51	0.55–4.15	0.424
Heart failure	1.17	0.15–8.88	0.881
Chronic kidney disease	1.11	0.26–4.74	0.887

Reference categories: SHR tertile 1 (≤0.873); NIHSS group mild (0–7); male sex; all other covariates absent. SHR was calculated using the Roberts formula: estimated average glucose (mmol/L) = (1.59 × HbA1c%) – 2.59, with SHR defined as admission glucose ÷ estimated average glucose. Linear trend across tertiles: odds ratio per tertile 1.90, *p*-trend = 0.002 (unadjusted). This analysis is a sensitivity check on the binary >7.8 mmol/L exposure used in **Table 3**, section A; the directional finding is preserved.

CI, confidence interval; mRS, modified Rankin Scale; NIHSS, National Institutes of Health Stroke Scale; OR, odds ratio; SHR, stress hyperglycemia ratio.

### Glycated hemoglobin and identification of occult diabetes

Among the 91 patients with no prior diagnosis of diabetes, HbA1c was documented in 76 (83.5%). Ofthose tested, 14 (18.4%) had HbA1c ≥6.5% (48 mmol/mol), consistent with previouslyundiagnosed diabetes, and a further 37 (48.7%) had HbA1c in the prediabetes range (5.7%–6.4%); these counts are summarized in **Supplementary Table 6**. This pattern was particularly marked in the subgroup of 18 patients with admission hyperglycemia but no historical diabetes diagnosis (initially classified as stress-induced hyperglycemia). Of the 15 patients with HbA1c documented, nine (60.0%) had HbA1c ≥6.5% (48 mmol/mol) and only two (13.3%) had genuinely normal chronic glycemia, indicating that the stress hyperglycemia label was misclassified in the majority of testable cases (**Table 1**).

In patients with established diabetes, the median HbA1c was 8.3% (interquartile range 7.0–10.1), substantially above the American Diabetes Association’s recommended pre-admission target of below 7.0% (25), indicating poor chronic glycemic control in this subgroup; the corresponding median in the no-known-diabetes group was 5.8% (IQR 5.6–6.2; Mann–Whitney *p* < 0.001). The pre-admission glycemic burden carried into the stroke unit by the subgroup with diabetes helps contextualize both the high inappropriate management rate and SHR findings reported above.

### In-hospital glucose trajectory

**Figure 3:**
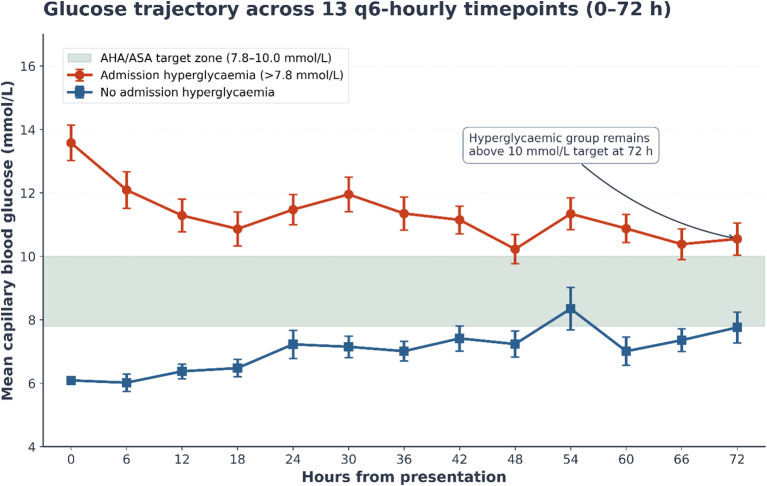
Capillary blood glucose trajectory from presentation to 72 h post-admission. The figure shows capillary blood glucose trajectory across 13 q6h timepoints (presentation to 72 h), stratified by admission hyperglycemia status. Mean ± standard error capillary blood glucose at each timepoint. The shaded band marks the American Heart Association/American Stroke Association in-hospital target zone of 7.8 to 10.0 mmol/L. The hyperglycemic group remained near or above the upper target (10 mmol/L) throughout the 72-h observation period; the euglycemic group remained within target throughout. AHA/ASA, American Heart Association/American Stroke Association.

**Figure 4:**
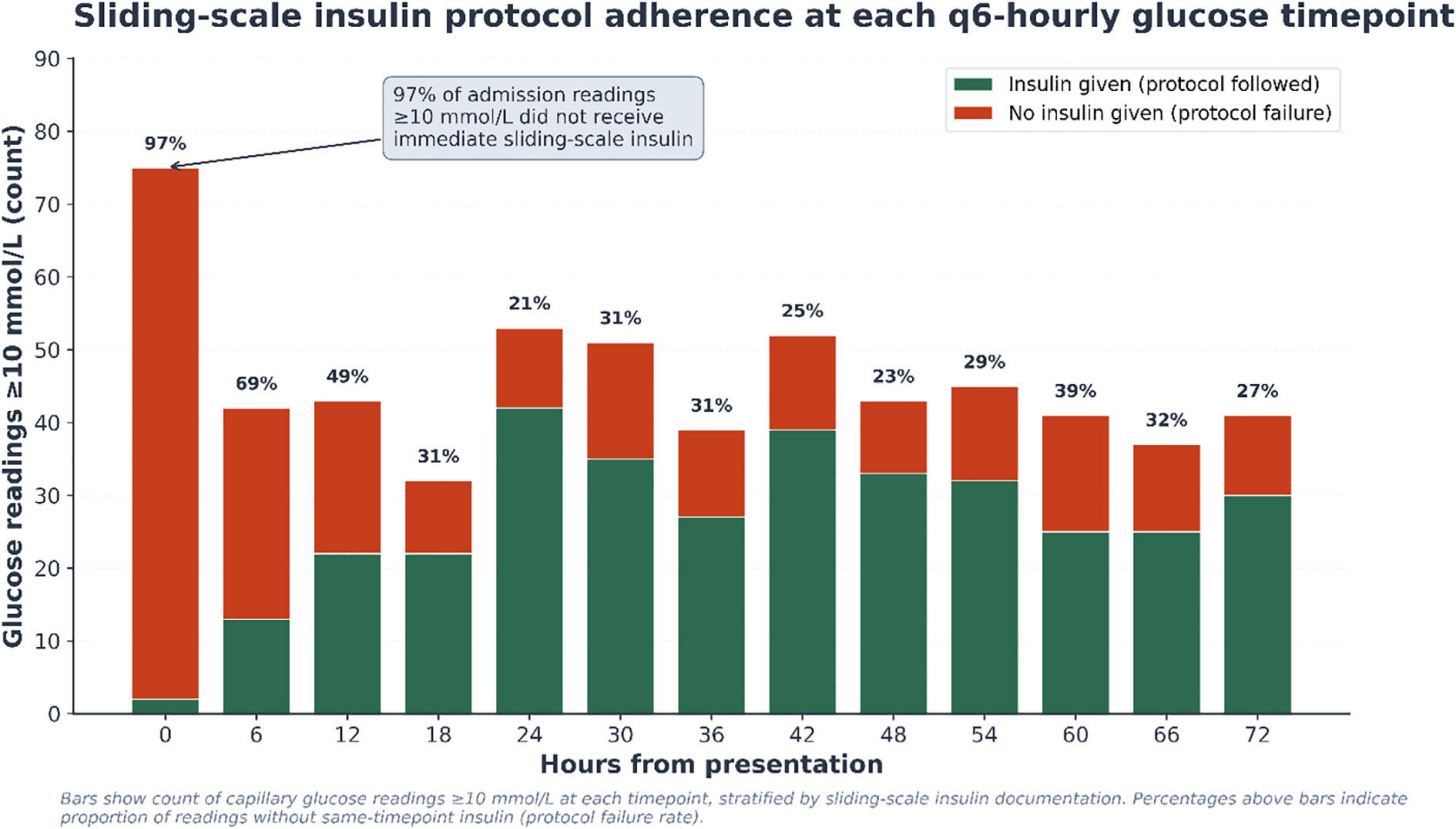
Sliding-scale insulin protocol adherence at each q6h timepoint. The stacked bars show the count of capillary glucose readings ≥10 mmol/L at each timepoint, partitioned by sliding-scale insulin documentation. The percentages above bars indicate the proportion of glucose readings ≥10 mmol/L that were not matched by same-timepoint sliding-scale insulin documentation (the algorithm-defined protocol failure rate).

Across the pre-specified 13 timepoints starting from the patients’ first presentation to 72 h post-presentation (**Figure 3**), the euglycemic group maintained a mean glucose level between 6.0 and 8.3 mmol/L, remaining below the SHINE-aligned upper target of 10 mmol/L throughout admission. The hyperglycemic group had a mean initial glucose level of 13.6 mmol/L, which improved gradually during admission but remained near or above the 10 mmol/L threshold throughout the admission period, with a mean of 10.5 mmol/L at 72 h. The proportion of hyperglycemic patients achieving glucose levels below 10 mmol/L increased from 29.2% at presentation to a fluctuating range of 35%–55% during follow-up, with only 52.1% below the target at 72 h (**Figure 4**). This persistent failure to reach the SHINE-aligned upper bound is consistent with the high algorithm-derived inappropriate management rate and quantifies the in-hospital glycemic burden missed by routine subcutaneous insulin protocols at our center.

### Effect modification by reperfusion therapy

**Figure 5:**
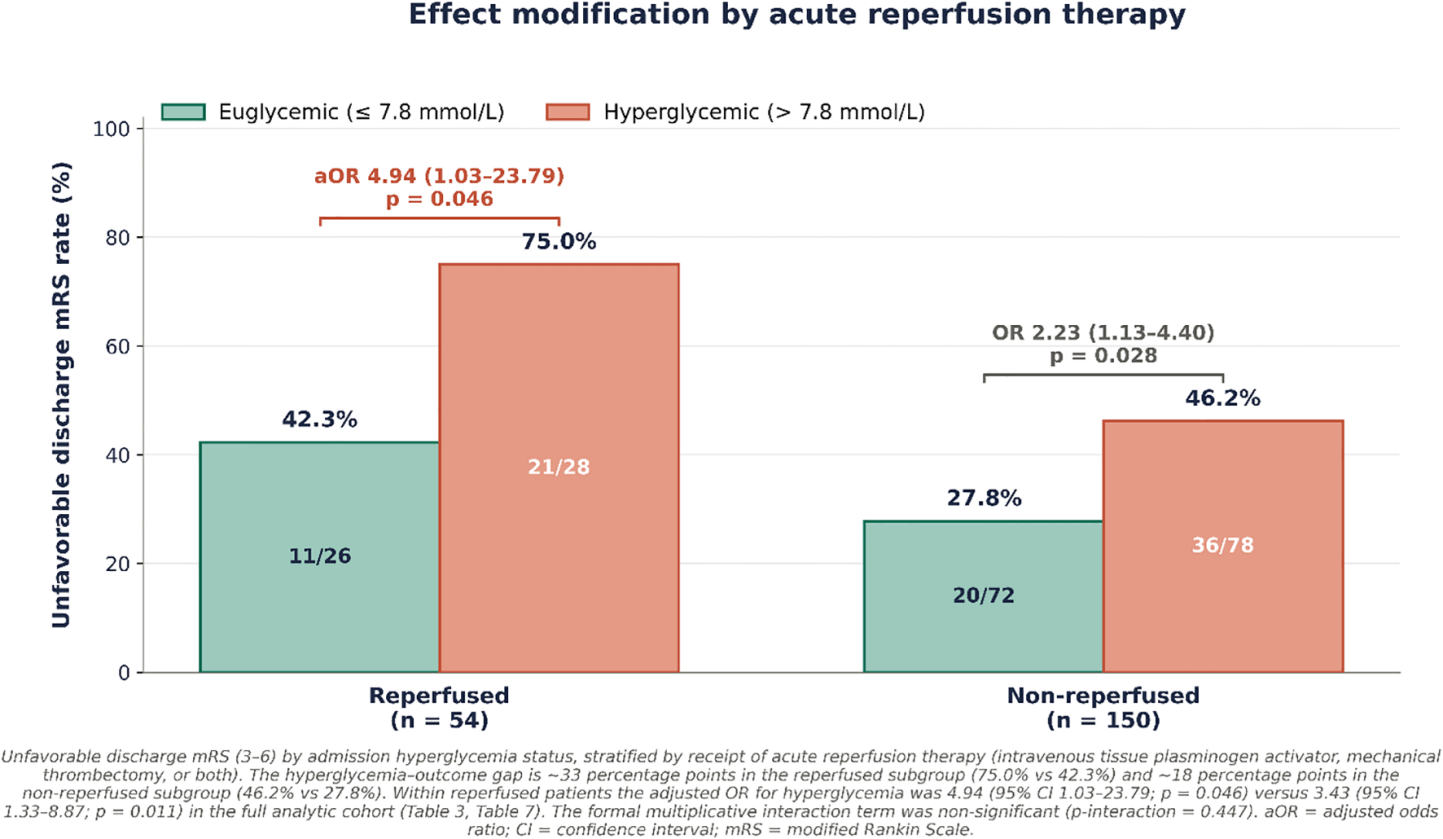
Effect modification in patients receiving acute reperfusion therapy. Unfavorable discharge mRS (3–6) rate by admission hyperglycemia status (>7.8 versus ≤7.8 mmol/L), stratified by receipt of acute reperfusion therapy (intravenous tissue plasminogen activator, mechanical thrombectomy, or both). Among patients receiving reperfusion therapy (*n* = 54), unfavorable discharge mRS occurred in 75.0% (21/28) of hyperglycemic patients compared with 42.3% (11/26) of euglycemic patients (adjusted OR 4.94, 95% CI 1.03–23.79; *p* = 0.046; **Table 5**). Among patients not receiving reperfusion therapy (*n* = 150), the corresponding rates were 46.2% (36/78) versus 27.8% (20/72), with a crude OR of 2.23 (95% CI 1.13–4.40; *p* = 0.028). The directional finding identifies patients receiving acute reperfusion therapy as a high-priority target population for future glycemic management interventions. aOR, adjusted odds ratio; CI, confidence interval; mRS, modified Rankin Scale; OR, odds ratio.

Among the 54 patients receiving acute reperfusion therapy with documented admission glucose, unfavorable discharge mRS occurred in 21 of 28 hyperglycemic patients (75.0%) compared with 11 of 26 euglycemic patients (42.3%; crude OR 4.09, 95% confidence CI 1.29–13.00, *p =* 0.026). After multivariable adjustment within the reperfusion therapy subgroup, the adjusted OR for admission hyperglycemia was 4.94 (95% confidence CI 1.03–23.79, *p =* 0.046; **Table 5**). The formal interaction term between admission hyperglycemia and reperfusion therapy was non-significant (*p*-interactio*n* = 0.447); however, the direction of the effect suggested that hyperglycemia may be particularly detrimental in patients undergoing acute reperfusion therapy (**Figure 5**). Inappropriate glycemic management was equally common in patients receiving and not receiving reperfusion therapy (47.3% versus 47.9%, *p* = 1.000), indicating that protocol failure is not explained by acuity-based resource triage.

**Table 5:** Adjusted multivariable logistic regression model for unfavorable discharge mRS within the reperfusion therapy subgroup.

Covariate	Adjusted OR	95% CI	*p*-value
Admission hyperglycemia (>7.8 mmol/L)	4.94	1.03–23.79	0.046
Age (per year)	1.04	0.98–1.10	0.197
Female sex	0.81	0.16–4.06	0.795
NIHSS group: moderate (8–14)	3.10	0.45–21.39	0.252
NIHSS group: severe (≥15)	26.20	2.46–278.73	0.007
Diabetes mellitus	0.86	0.18–4.09	0.846

Adjusted analysis restricted to patients receiving any acute reperfusion therapy (intravenous tissue plasminogen activator, mechanical thrombectomy, or both) with documented admission glucose; *n* = 54, 32 events. Reference categories: NIHSS group mild (0–7); male sex.

CI, confidence interval; NIHSS, National Institutes of Health Stroke Scale; OR, odds ratio.

### Effect modification by time to presentation

The last known well to ED arrival was documented in 182 of 218 patients (83.5%); 154 (84.6%)presented within 24 h, and 28 (15.4%) presented later. Time-to-presentation was not associatedwith discharge mRS (late versus early crude OR 1.13, 95% confidence interval [CI] 0.50–2.56, *p =* 0.835) or in-hospital death (0.91, 0.11–7.89, *p =* 1.000), and the addition of time-to-presentation to the primary multivariable model did not attenuate the association between admission hyperglycemia (**Supplementary Table 7**). The interaction between admission hyperglycemia and time to presentation was not significant (*p* = 0.295). Within the early-presenter subgroup (*n* = 131, eligible for time-sensitive reperfusion therapy or active glycemic management interventions), the adjusted OR for admission hyperglycemia was 8.39 (95% confidence CI 2.26–31.20, *p* = 0.002). Within the smaller late-presenter subgroup (*n* = 21), the adjusted estimate was unstable owing to the small number of patients (crude OR 2.67, 95% CI 0.43–16.39, Fisher *p* = 0.387). The principal admission-hyperglycemia association therefore extends to the patient population most likely to benefit from active in-hospital glycemic intervention.

### Exploratory secondary outcome: 90-day mRS

The 90-day mRS was documented in only 53 patients (24.3% of the cohort), reflecting the limitations of post-discharge follow-up in routine practice. In this exploratory subset, 25 patients (47.2%) had unfavorable 90-day outcomes; the crude OR for admission hyperglycemia was 6.14 (95% confidence CI 1.58–28.16, Fisher’s exact *p* = 0.004), which was directionally consistent with the discharge analysis. Multivariate analysis was not performed because of the small subset and event counts. Given the substantial loss to follow-up, the 90-day mRS was retained as an exploratory secondary outcome only and was not subject to Bonferroni adjustment with the two clinical co-primary outcomes; discharge mRS, which is uniformly available, was the principal functional endpoint of this study.

## Discussion

In this first dedicated analysis of hyperglycemia and stroke outcomes from the United Arab Emirates, admission capillary blood glucose >7.8 mmol/L was independently associated with approximately 3.4-fold higher adjusted odds of unfavorable discharge functional status (adjusted OR 3.43, 95% CI 1.33–8.87) after adjustment for stroke severity, demographics, and the pre-specified nine-covariate set. The association was preserved after Mantel–Haenszel pooling across NIHSS strata (pooled OR 3.47); the model demonstrated excellent calibration (Hosmer–Lemeshow *p* = 0.873), and there was no concerning multicollinearity. Patients with admission hyperglycemia were also overwhelmingly more likely to receive non-adherent in-hospital glycemic management (85.8% vs. 11.1%), establishing hyperglycemia at presentation as both an outcome associated factor and the most informative clinical marker of an actionable implementation gap. The supplementary stress hyperglycemia ratio analysis reproduced the dose–response pattern in crude estimates (unfavorable mRS rising from 27% in the lowest tertile to 56% in the highest), with attenuation after adjustment for diabetes and NIHSS severity. Severe hypoglycemia did not occur in any patient under the predominantly subcutaneous insulin pathway, reinforcing the safety profile demonstrated in the standard arm of the SHINE trial (9).

The magnitude of the observed adjusted OR for discharge mRS was consistent with recent European and East Asian observational studies. Rinkel et al. reported an adjusted common OR of 1.69 for a shift toward worse 90-day mRS in a registry of endovascular thrombectomy patients with admission glucose above 7.8 mmol/L (23). Climent et al., working in the Hospital del Mar Basicmar registry, demonstrated an OR of 1.88 for 3-month mortality in the highest acute-to-chronic glycemic ratio tertile (24). Among studies using the stress hyperglycemia ratio, Merlino et al. reported OR values up to 4.52 for poor 3-month outcome in patients who underwent mechanical thrombectomy (25), and a recent dose–response meta-analysis of cohort studies reported a pooled OR of 2.64 (95% confidence CI 2.05–3.41) for poor functional outcome with an elevated SHR (26). Our adjusted estimate (3.43) sits within the upper portion of this range and likely reflects the high proportion of established diabetes and elevated HbA1c levels in the hyperglycemic group (median, 8.6%) and a relatively well-controlled comparator group. Directional consistency across the international literature and our own SHR tertile gradient indicates that the relationship between admission glycemia and outcome is reproducible, biologically graded, and clinically relevant to patients with stroke.

The implementation gap reported here is the first quantitative description of adherence to glycemic management in a stroke unit in the Gulf region. Approximately half of all admissions met at least one algorithm-defined SHINE-aligned protocol failure criterion, and the failure rate was concentrated in patients with established diabetes and elevated presenting glucose levels, that is, the patients who most needed structured insulin pathways. The pattern echoes earlier findings from US cohorts showing undertreatment of in-hospital hyperglycemia in patients with known diabetes (9) but extends them to a population with one of the world’s highest diabetes prevalence rates, where the absolute number of affected admissions is large (14, 27). The algorithm captures discrete protocol failure events at specified glucose–insulin pairs in a transparent and reproducible manner, allowing other Gulf centers to apply the same definition to their datasets.

The adjusted odds ratio for receiving reperfusion therapy in the primary mRS model (1.47, 95% CI 0.56–3.85, *p* = 0.433) warrants brief comment. The reperfusion therapy group at this center was heterogeneous: of the 54 reperfused patients with documented admission glucose, 23 (43%) received intravenous thrombolysis only, 19 (35%) received mechanical thrombectomy only, and 12 (22%) received both. Mechanical thrombectomy was preferentially selected for severe large vessel occlusion strokes, while thrombolysis alone tended to be used for milder deficits within the time window. The non-significant directionally adverse main effect is most plausibly explained by confounding by indication: the small reperfusion therapy subgroup contained a non-random mix of stroke severities that the NIHSS group could not fully resolve. This finding should not be interpreted as evidence that reperfusion therapy is ineffective at this center, but rather as a limitation of single-center observational analysis. The within-subgroup effect modification by admission hyperglycemia (adjusted OR 4.94 versus 3.43 in the full cohort; **Figure 5**, **Table 5**) was unaffected by this confounding and is the more informative finding. The biological mechanism is plausible: hyperglycemia exacerbates reperfusion injury, accelerates blood–brain barrier disruption, and increases hemorrhagic transformation risk, identifying a high-priority target population for future glycemic management. We use “reperfusion therapy” rather than “reperfused” because vessel recanalization status (thrombolysis in cerebral infarction—TICI score 2b/3) was not systematically documented and could not be confirmed for all treated patients. The principal hyperglycemia–outcome association was preserved within the early-presenter subgroup eligible for reperfusion therapy (adjusted OR 8.39, 95% CI 2.26–31.20), with time to presentation neither modifying nor confounding the effect.

The mortality findings should be interpreted with caution because of the low overall event rate in this study. The crude and adjusted ORs were consistently above 3.5 in every analytical model (cross-product crude 3.71, parsimonious adjusted 5.61), but with wide confidence intervals reflecting only 14 deaths in the descriptive cohort and seven events available to the complete-case parsimonious model (**Table 3**). While the directional evidence is concordant with mortality findings from larger cohorts and meta-analyses (12, 19, 26), confirmation of an independent mortality effect in this population requires a larger Gulf cohort or registry pooled analysis. The underpowered nature of this cohort for the mortality endpoint and the corresponding sample size implications are addressed in the “Strengths and limitations” section. The 90-day mRS subgroup was similarly limited by loss to follow-up (24.3% retention), reflecting a persistent challenge in post-discharge stroke surveillance regionally (16, 17). The directional consistency with the discharge analysis (crude OR 6.14) supports the inference that the discharge mRS finding extends to medium-term functional outcomes.

Several mechanistic explanations support this association. Hyperglycemia accelerates oxidative injury and lactate accumulation in the ischemic penumbra, impairs collateral circulation, exacerbates blood–brain barrier disruption, and increases the likelihood of hemorrhagic transformation after reperfusion (3, 5, 7). Proportional injury appears more pronounced in patients without diabetes who have stress hyperglycemia, where the relative excursion above chronic glucose levels is greater, accounting for the dose–response findings reported with stress hyperglycemia and acute-to-chronic glycemic ratios (6, 7, 20–22). From an implementation perspective, the absence of severe hypoglycemia under our current pathway is reassuring and supports the SHINE-recommended subcutaneous insulin approach with a target of 7.8–10.0 mmol/L (9, 10). This subcutaneous pathway matches the standard arm of the SHINE trial; our center does not routinely use the continuous intravenous insulin infusion of the SHINE intensive arm, which targeted 4.4–7.2 mmol/L and did not improve the 90-day mRS while increasing the hypoglycemia risk (9). The SHINE result therefore supports the safety and adequacy of the subcutaneous pathway used at our center. The implication for our region and local practice is that intensifying glycemic monitoring and protocolizing sliding-scale insulin escalation can be performed without any significant hypoglycemia risk under the existing inpatient subcutaneous insulin pathway.

### Clinical and quality improvement implications

These findings have three practical implications for managers. First, admission glucose levels should be measured and documented in every acute ischemic stroke admission, with q6h capillary monitoring continued for 72 h in any patient with admission glucose >7.8 mmol/L or known diabetes, in line with the 2019 American Heart Association/American Stroke Association guidelines (10). Second, a documented glycemic target (7.8 to 10.0 mmol/L) and a written sliding-scale insulin protocol with explicit dose-escalation rules for repeat readings at or above 10 mmol/L should be incorporated into the standard stroke admission order set; this addresses the most common protocol failure observed in the present study. Third, given the very high prevalence of diabetes among Gulf stroke patients (13, 14, 27, 28), routine HbA1c testing at admission is justified to identify both undiagnosed diabetes and patients at the greatest risk of management deviation and would also enable routine SHR calculation for prognostic stratification. These steps are low-cost and process-level rather than therapy-level and can be delivered within existing stroke unit workflows.

The implementation gap demonstrated in this study reflects a wider disconnect between acute stroke management and contemporary inpatient diabetes care. The 2019 American Heart Association/American Stroke Association guidelines specify an in-hospital glycemic target of 7.8 to 10.0 mmol/L (10) but defer to endocrine guidelines on how to achieve this. The American Diabetes Association Standards of Care in Diabetes Section 16 explicitly states that the sole use of sliding-scale insulin in the inpatient setting is strongly discouraged, and it recommends weight-based basal-bolus regimens (typically 0.3 to 0.4 units/kg/day) for any hospitalized patient with established or newly identified diabetes (29). The RABBIT 2 randomized trial demonstrated that basal-bolus regimens achieve substantially better glycemic control than sliding-scale insulin alone in hospitalized patients with diabetes without increasing hypoglycemia (30). The clinical case for incorporating routine admission HbA1c testing into Gulf stroke admission order sets is reinforced by the data presented in **Table 1**: 14 of 76 patients not known to have diabetes and tested in this cohort (18.4%) had pre-existing diabetes, and a further 37 (48.7%) had prediabetes, with the further detail that nine of 15 patients initially classified as having “stress hyperglycemia” were actually having occult diabetes on HbA1c testing. This single inexpensive test allows immediate triage between true stress hyperglycemia, where reactive correction may suffice, and chronic hyperglycemia, where ADA-compliant proactive basal-bolus pathways are required.

### Strengths and limitations

This study had several strengths. This is the first observational analysis of hyperglycemia in acute ischemic stroke in the United Arab Emirates and the first quantitative audit of SHINE-aligned adherence to glycemic management in a Gulf stroke unit. The fully multivariable adjustment set included nine clinically relevant confounding factors. The primary threshold was aligned with the American Heart Association/American Stroke Association recommendations, allowing direct comparisons with international cohorts. Bonferroni correction was applied across the two clinical co-primary outcomes (*α* = 0.025), and Mantel–Haenszel stratification confirmed that the principal finding was not driven by stroke severity strata. A sensitivity analysis with NIHSS as a continuous covariate produced near-identical estimates and substantially better statistical fit (ΔAIC 21.9), confirming insensitivity to NIHSS coding. The supplementary SHR analysis demonstrated a clear unadjusted dose–response and positioned the findings within the contemporary literature on relative versus absolute hyperglycemia.

The principal limitations are the retrospective single-center design, modest mortality event count (14 deaths), substantial loss to 90-day follow-up (75.7%), and absence of information on infarct volume or onset-to-presentation time at the imaging level. The mortality finding requires additional interpretation given its borderline crude significance (Fisher’s exact test *p =* 0.051) and concordance with the wider literature. The cohort was underpowered for this outcome at conventional significance thresholds, with a *post-hoc* power of 59.1% at *α* = 0.05 and 48.4% at the Bonferroni-corrected *α* = 0.025; an *a priori* sample size of approximately 181 patients per group would have been required to confirm the observed effect with 80% power. The directional association between admission hyperglycemia and in-hospital death (adjusted OR 5.61) is consistent with that of prior cohort studies (1, 2, 7); however, it cannot be confirmed at conventional significance thresholds in this single-center cohort. A larger Gulf-region multicenter cohort is required to definitively test this association and assess whether the management gap demonstrated here generalizes to other UAE and regional centers.

The algorithm-derived inappropriate management metric is sensitive to documentation completeness and may overstate failure if sliding-scale insulin is administered but not recorded at the matched glucose time-point. The SHR analysis was restricted to 166 patients who had both admission glucose and HbA1c documented, and 52 patients were excluded due to lack of these data. Routine admission HbA1c testing in future cohorts could mitigate this limitation. No severe hypoglycemia was documented in any of the patients in this study, which is reassuring but limits inferences about hypoglycemia risk under alternative protocols. The generalizability of our findings beyond Tawam Hospital Stroke Centre and the predominantly male, South Asian, and Emirati patient mix remains uncertain and requires further multicenter large-sample studies. Volumetric neuroimaging metrics, including the Alberta Stroke Program Early CT Score (ASPECTS) (31), and infarct volume in milliliters were documented in only 10 (4.6%) and 13 (6.0%) patients, respectively. At Tawam Hospital, stroke etiology is routinely characterized in the clinical chart through combined imaging and clinical assessment (cardio-embolic, intracranial atherosclerotic, lacunar, arterial dissection, or vasculitic mechanisms), with CT head as one of several inputs. However, this clinical etiologic classification was recorded as free text within radiology conclusion fields and clinical notes rather than as discrete structured data elements; therefore, we were unable to extract formal Trial of Org 10172 in Acute Stroke Treatment (TOAST) categories for multivariable adjustment in this retrospective dataset (32). Although the National Institutes of Health Stroke Scale group is a validated clinical proxy for stroke severity, future Gulf cohorts incorporating volumetric imaging and TOAST classification would allow more precise isolation of the metabolic association of stress hyperglycemia from the volume of ischemic tissue injury.

Several other recognized confounders of the hyperglycemia–outcome association were not available for analysis. We did not have access to fully assess for the volumetric infarct measurement, formal Alberta Stroke Program Early CT Score (ASPECTS) scoring, or recanalization grade (TICI score) for the reperfusion therapy subgroup. We also did not have systematic documentation of premorbid frailty (modified Rankin pre-admission was captured in only 4.6% of patients), in-hospital infection or sepsis, and exogenous steroid exposure, all of which can independently elevate inpatient glucose levels and may confound the observed association. The directional finding therefore reflects an association rather than a causal effect; admission hyperglycemia in this cohort may, in part, represent a marker of severe physiological stress rather than an independent mediator of poor outcome.

Another limitation is that our cohort demographics differ from those typically reported in Western stroke registries. The mean patient age was approximately 58 years, lower than the 65–75-year range typical of European and North American cohorts, and the female proportion was 22.9%, lower than the approximate 50% reported in most international stroke registries. These differences reflect the underlying demographic structure of the United Arab Emirates population, which has a substantial working-age male expatriate component contributing disproportionately to acute stroke admissions, and a documented earlier age of cardiometabolic disease onset across the Gulf Cooperation Council region (13, 14). The generalizability of our findings to populations with a different demographic mix should be considered with this regional context in mind.

## Conclusions

Admission hyperglycemia >7.8 mmol/L was independently associated with approximately 3.4-fold adjusted odds of unfavorable discharge functional status and identified the subset of acute ischemic stroke patients most likely to receive non-adherent in-hospital glycemic management at a tertiary stroke center in the United Arab Emirates. The directional association with in-hospital death (adjusted OR 5.61) was concordant with international evidence but was underpowered for definitive confirmation in this single-center cohort study. Stress hyperglycemia ratio analysis demonstrated a graded crude dose–response that attenuated after adjustment for diabetes and stroke severity, indicating that admission glucose, chronic glycemic state, and the relative excursion between them each carry prognostic information. The complete absence of severe hypoglycemia under the predominantly subcutaneous insulin pathway supports the safety profile of SHINE-aligned standard care. The implementation gap can be closed through structured admission orders, documented targets, q6h glucose monitoring, and protocolized sliding-scale insulin administration. These data establish a benchmark for stroke services in the Gulf region and identify a concrete, low-cost, quality improvement target that should be evaluated in a multicenter regional collaboration.

## Data Availability

The de-identified analytic dataset and statistical analysis syntax are available from the corresponding author upon reasonable request, subject to institutional data-sharing approval and a data-use agreement.
