# Supplementary material for: Admission hyperglycemia, in-hospital glycemic management, and discharge outcomes in acute ischemic stroke: a UAE comprehensive stroke center cohort
Source: Front Endocrinol (Lausanne). 2026 Jul 15;17:1887754. doi: 10.3389/fendo.2026.1887754 (PMC13414823; doi:10.3389/fendo.2026.1887754)

**Supplementary File**

# Admission hyperglycemia, in-hospital glycemic management, and discharge outcomes in acute ischemic stroke: a UAE comprehensive stroke center cohort

**Supplementary Tables**

***Supplementary Table S1. Comparison of patients with missing versus documented admission glucose***

| **Variable** | **Missing Adm CBG (n=13)** | **Documented (n=205)** | **p-value** |
|---|---|---|---|
| Age, years, median [IQR] | 50 [47–61] | 56 [48–69] | 0.607 |
| NIHSS, median [IQR] | 4 [2–6] | 4 [2–8] | 0.566 |
| Length of stay, days, median [IQR] | 8 [5–14] | 5 [4–10] | 0.078 |
| Female sex, n (%) | 1 (7.7) | 49 (23.9) | 0.307 |
| Diabetes mellitus, n (%) | 4 (30.8) | 123 (60.0) | 0.046 |
| Atrial fibrillation, n (%) | 1 (7.7) | 18 (8.8) | 1.000 |
| Previous stroke / TIA, n (%) | 4 (30.8) | 40 (19.5) | 0.303 |
| Heart failure, n (%) | 1 (7.7) | 17 (8.3) | 1.000 |
| Chronic kidney disease, n (%) | 2 (15.4) | 28 (13.7) | 0.695 |
| Reperfusion therapy, n (%) | 1 (7.7) | 54 (26.3) | 0.192 |
| In-hospital death, n (%) | 0 (0.0) | 14 (6.8) | 1.000 |
| Unfavorable discharge mRS, n (%) | 7 (53.8) | 88 (42.9) | 0.566 |
| NIHSS severe (≥15), n (%) | 0/10 (0.0) | 26/174 (14.9) | 0.362 |

*Continuous variables: Mann-Whitney U test. Categorical variables: Fisher's exact test. Adm CBG= admission capillary blood glucose; mRS = modified Rankin Scale; NIHSS = National Institutes of Health Stroke Scale; TIA = transient ischemic attack.*

*Supplementary Table S2. Firth penalized logistic regression — sensitivity analysis for the three primary multivariable models*

**Panel A. Primary outcome: Unfavorable discharge modified Rankin Scale (mRS 3–6); complete-case n = 173, 70 events.**

| **Covariate** | **Unpenalized aOR (95% CI), p** | **Firth aOR (95% CI), p** |
|---|---|---|
| Admission hyperglycemia (>7.8 mmol/L) | 3.43 (1.33–8.87), p=0.011 | 3.10 (1.25–7.71), p=0.015 |
| Age (per year) | 1.02 (0.99–1.05), p=0.106 | 1.02 (0.99–1.05), p=0.135 |
| Female sex | 1.27 (0.51–3.16), p=0.601 | 1.24 (0.51–3.00), p=0.638 |
| NIHSS moderate (8–14) | 4.95 (1.64–14.98), p=0.005 | 4.31 (1.46–12.71), p=0.008 |
| NIHSS severe (≥15) | 20.91 (4.90–89.17), p<0.001 | 14.38 (3.77–54.82), p<0.001 |
| Diabetes mellitus | 1.26 (0.45–3.54), p=0.655 | 1.24 (0.46–3.34), p=0.667 |
| Prior stroke / TIA | 1.38 (0.53–3.58), p=0.505 | 1.37 (0.54–3.46), p=0.513 |
| Atrial fibrillation | 0.45 (0.08–2.63), p=0.373 | 0.49 (0.09–2.66), p=0.407 |
| Reperfusion therapy | 1.47 (0.56–3.85), p=0.433 | 1.46 (0.57–3.70), p=0.429 |
| Heart failure | 1.42 (0.29–6.89), p=0.667 | 1.36 (0.29–6.34), p=0.692 |
| Chronic kidney disease | 0.74 (0.19–2.91), p=0.663 | 0.76 (0.20–2.88), p=0.692 |

**Panel B. In-hospital death (parsimonious 3-covariate model); complete-case n = 174, 7 events.**

| **Covariate** | **Unpenalized aOR (95% CI), p** | **Firth aOR (95% CI), p** |
|---|---|---|
| Admission hyperglycemia (>7.8 mmol/L) | 5.61 (0.65–48.43), p=0.117 | 3.76 (0.70–20.25), p=0.123 |

| | | |
|---|---|---|
| Age (per year) | 1.03 (0.98–1.09), p=0.256 | 1.03 (0.98–1.08), p=0.240 |
| NIHSS moderate (8–14) | 3.71 (0.56–24.63), p=0.175 | 3.83 (0.71–20.71), p=0.119 |
| NIHSS severe (≥15) | 2.49 (0.36–17.15), p=0.355 | 2.68 (0.49–14.77), p=0.257 |

**Panel C. Algorithm-derived inappropriate glycemic management; complete-case n = 174, 84 events.**

| Covariate | Unpenalized aOR (95% CI), p | Firth aOR (95% CI), p |
|---|---|---|
| Admission hyperglycemia (>7.8 mmol/L) | 80.59 (20.21–321.4), p<0.001 | 53.75 (15.66–184.49), p<0.001 |
| Diabetes mellitus | 45.38 (8.79–234.4), p<0.001 | 31.47 (7.16–138.34), p<0.001 |
| NIHSS moderate (8–14) | 3.90 (0.56–27.25), p=0.170 | 3.29 (0.54–20.19), p=0.199 |
| NIHSS severe (≥15) | 2.11 (0.47–9.38), p=0.328 | 1.94 (0.46–8.14), p=0.363 |
| Age (per year) | 1.00 (0.96–1.04), p=0.980 | 1.00 (0.96–1.04), p=0.955 |
| Female sex | 0.16 (0.03–0.77), p=0.023 | 0.18 (0.04–0.79), p=0.023 |

*Firth penalized logistic regression performed using Python 3 (scipy 1.11, statsmodels 0.14) with a Jeffreys prior penalty as described by Firth [33] and Heinze and Schemper [34]. Firth's penalty modifies the score function with a term that produces finite estimates under separation and reduces small-sample bias. aOR = adjusted odds ratio; CI = confidence interval; NIHSS = National Institutes of Health Stroke Scale; TIA = transient ischemic attack.*

***Supplementary Table S3. Sensitivity analysis: crude and adjusted ORs for the two clinical co-primary outcomes across three pre-specified admission-glucose threshold definitions.***

| Definition | n / events (CC) | Crude OR (95% CI) | Adjusted OR (95% CI) | p (adj.) |
|---|---|---|---|---|
| **Outcome 1: Unfavorable discharge mRS (3–6)** | | | | |
| Def A: split-protocol (>6.1 if DM; >8.3 if no DM) | 173 / 70 | 2.26 (1.26–4.04) | 2.09 (0.63–6.88) | 0.227 |
| **Def B: >7.8 mmol/L (PRIMARY)** | **173 / 70** | **2.51 (1.42–4.45)** | **3.43 (1.33–8.87)** | **0.011** |
| Def C: >10 mmol/L | 173 / 70 | 2.19 (1.23–3.92) | 2.14 (0.89–5.14) | 0.088 |
| **Outcome 2: In-hospital death (parsimonious model)** | | | | |
| Def A: split-protocol | 174 / 7 | 4.71 (1.03–21.62) | 4.19 (0.48–36.35) | 0.194 |

| | | | | |
|---|---|---|---|---|
| **Def B: >7.8 mmol/L (PRIMARY)** | **174 / 7** | **3.71 (1.00–13.70)** | **5.61 (0.65–48.43)** | **0.117** |
| Def C: >10 mmol/L | 174 / 7 | 2.53 (0.84–7.58) | 4.21 (0.78–22.76) | 0.095 |

*Three pre-specified admission-glucose threshold definitions were tested as a sensitivity analysis of exposure specification. Def A (split-protocol cutoff): admission capillary blood glucose >6.1 mmol/L if known diabetes mellitus, or >8.3 mmol/L if no known diabetes. Def B (PRIMARY): admission capillary blood glucose >7.8 mmol/L (American Heart Association/American Stroke Association in-hospital target lower bound). Def C: admission capillary blood glucose >10 mmol/L. Estimates are directionally consistent across all three threshold definitions for both co-primary outcomes. Adjusted ORs for unfavorable discharge mRS use the full nine-covariate model (Table 3); adjusted ORs for in-hospital death use the parsimonious three-covariate model (Table 4). CC = complete-case sample; CI = confidence interval; DM = diabetes mellitus; mRS = modified Rankin Scale; OR = odds ratio.*

***Supplementary Table S4. Algorithm-defined inappropriate-management episodes, by failure type (cohort total).***

| Failure type | Algorithmic definition | Episodes (n) | % of inappropriate episodes | Patients with ≥1 episode of this type, n (% of cohort) |
|---|---|---|---|---|
| **Denominators** | | | | |
| Glucose readings (cohort total) | All measured capillary glucose values across the 13 pre-specified q6-hourly timepoints | 1,506 | — | — |

| | | | | |
|---|---|---|---|---|
| | (presentation to 72 h) in 218 patients | | | |
| Readings with glucose ≥ 10 mmol/L | Same as above, restricted to readings exceeding the AHA/ASA in-hospital upper target | 594 (39.4% of 1,506) | — | — |
| Total inappropriate-management episodes | Sum of failure types (a) + (b) below | 260 (43.8% of 594) | 100.0% | 104 (47.7%)* |
| **Inappropriate-management episodes by failure type** | | | | |
| (a) Hyperglycemia without insulin response | Capillary glucose ≥ 10 mmol/L without same-timepoint sliding-scale insulin documentation | 247 | 95.0% | 97 (44.5%) |

| | | | | |
|---|---|---|---|---|
| (b) Mild hypoglycemia | Capillary glucose < 4 mmol/L at any q6-hourly timepoint | 13 | 5.0% | 7 (3.2%) |
| **Safety check** | | | | |
| Severe hypoglycemia (patient-level) | Patient-level minimum capillary glucose < 2.2 mmol/L during index admission (Table 1, section C) | 0 | — | 0 (0.0%) |

*Numerators are episode-level counts derived from patient-by-timepoint glucose and sliding-scale insulin documentation across the 13 pre-specified q6-hourly observation timepoints (presentation, 6 h, 12 h, 18 h, 24 h, 30 h, 36 h, 42 h, 48 h, 54 h, 60 h, 66 h, 72 h). *The patient-level inappropriate-management count (104 of 218; 47.7 per cent) is smaller than the simple sum of patients with at least one type (a) plus at least one type (b) episode because seven patients had episodes of both types and are counted once at the patient level. Type (a) is the dominant failure mode and accounts for 95.0 per cent of all inappropriate-management episodes; this proportion is the*

*basis for the Results-section statement and motivates the implementation recommendations summarized in the Graphical Abstract (structured admission orders, q6-hourly monitoring, documented sliding-scale escalation rules). Type (b) episodes were all in the mild hypoglycemia range (3.0 to 3.9 mmol/L); no patient met the severe hypoglycemia threshold (< 2.2 mmol/L) at the patient-level minimum, consistent with the safety profile reported in Table 1, section C. AHA/ASA = American Heart Association / American Stroke Association.*

***Supplementary Table S5. Univariate predictors of inappropriate glycemic management.***

| | | | |
|---|---|---|---|
| Diabetes mellitus, n (%) | 95 (91.3) | 32 (28.1) | <0.001 |
| Diabetes mellitus | n (%) | 95 (91.3) | 32 (28.1) |
| Admission glucose mmol/L median [IQR] | 12.1 [9.6–15.6] | 6.1 [5.4–6.9] | <0.001 |
| Highest in-hospital glucose mmol/L median [IQR] | 15.5 [11.4–18.8] | 7.4 [6.4–8.9] | <0.001 |
| HbA1c % median [IQR] | 8.6 [7.0–10.8] | 5.9 [5.6–6.4] | <0.001 |
| Age years median [IQR] | 60.0 [52.0–69.0] | 53.0 [44.0–65.0] | 0.007 |
| Reperfusion therapy received n (%) | 26 (25.0) | 28 (24.6) | 1.000 |
| Female sex n (%) | 24 (23.1) | 25 (21.9) | 0.949 |
| Severe NIHSS (≥15) n (%) | 12 (11.5) | 14 (12.3) | 0.783 |

*Univariate associations between baseline characteristics and the binary outcome of inappropriate glycemic management (≥1 episode meeting algorithm-derived criteria during the index admission). Continuous variables compared with Mann–Whitney U test; categorical variables with chi-square or Fisher's exact test as appropriate. HbA1c = glycated hemoglobin; IQR = interquartile range; NIHSS = National Institutes of Health Stroke Scale.*

***Supplementary Table S6. HbA1c category distribution among patients with no prior diagnosis of diabetes, stratified by admission glycemic status.***

| HbA1c category | Hyperglycemic at admission (n=15)* | Euglycemic at admission (n=53) | Total no-known-DM (n=76)† |
|---|---|---|---|
| Diabetes (HbA1c ≥6.5%) | 9 (60.0%) | 5 (9.4%) | 14 (18.4%) |
| Prediabetes (HbA1c 5.7–6.4%) | 4 (26.7%) | 33 (62.3%) | 37 (48.7%) |
| Normal (HbA1c <5.7%) | 2 (13.3%) | 15 (28.3%) | 25 (32.9%) |

**Of 18 patients with admission hyperglycemia and no prior diabetes diagnosis (initially classified as 'stress hyperglycemia'), HbA1c was documented in 15. †Of 91 patients with no prior diabetes diagnosis, HbA1c was documented in 76 (83.5%); 15 patients had HbA1c missing. Diabetes diagnostic threshold: HbA1c ≥6.5% per American Diabetes Association Standards of Care. Median HbA1c in known-DM group (n = 102 with HbA1c documented): 8.3% (IQR 7.0–10.1) versus 5.8% (IQR 5.6–6.2) in no-known-DM group; Mann–Whitney p < 0.001. DM = diabetes mellitus; HbA1c = glycated hemoglobin.*

***Supplementary Table S7. Time-to-presentation sub-analysis: hyperglycemia–outcome association with time as a covariate and within strata.***

| **Analysis** | **n** | **Events** | **Adjusted OR (95% CI)** | **p-value** |
|---|---|---|---|---|
| Primary model (Table 3 reference) | 173 | 70 | 3.43 (1.33–8.87) | 0.011 |
| *Primary model + time-to-presentation (152, 58 events)* | | | | |
| Admission hyperglycemia | | | 5.21 (1.72–15.76) | 0.003 |
| Late presentation (>24h) | | | 2.45 (0.76–7.91) | 0.133 |
| *Interaction model: hyperglycemia × time* | 152 | 58 | | |
| Multiplicative interaction term | | | 0.31 (0.03–2.81) | 0.295 |

| | | | | |
|---|---|---|---|---|
| Likelihood ratio test | | | — | 0.299 |
| *Stratified by time* | | | | |
| Early presenters (≤24 h) | 131 | 50 | 8.39 (2.26–31.20) | 0.002 |
| Late presenters (>24 h) | 21 | 8 | not estimable* | — |

**Crude OR for late presenters: 2.67 (95% CI 0.43–16.39, Fisher p = 0.387). Adjusted estimate not reported because the small subset (n = 21, 8 events) cannot support the nine-covariate adjustment under the events-per-variable rule. Time to presentation defined by the BE-FAST last-known-well to ED arrival categorization: late presentation = >24 h; early presentation = ≤24 h. 36 of 218 patients (16.5%) had no documented time of last known well and are excluded from this sub-analysis. All adjusted models used the same covariate set as the primary multivariable model in Table 3 plus time-to-presentation where indicated. BE-FAST = Balance, Eyes, Face, Arms, Speech, Time; CI = confidence interval; ED = emergency department; OR = odds ratio.*

## Supplementary Figure Legends

**Supplementary Figure S1. Receiver operating characteristic (ROC) curve for the primary multivariable model of unfavorable discharge mRS.**

Area under the curve = 0.814 (95 per cent confidence interval 0.746 to 0.879, derived from 1,000 bootstrap resamples). Dashed diagonal line indicates no discrimination. AUC = area under the curve; CI = confidence interval; mRS = modified Rankin Scale; ROC = receiver operating characteristic.

**Supplementary Figure S2. Stratified analysis of admission hyperglycemia by NIHSS group.**

Stratum-specific cross-product odds ratios with Wald 95 per cent confidence intervals for unfavorable discharge mRS, alongside the Mantel–Haenszel pooled estimate (green diamond). The hyperglycemia–outcome association is preserved across all three severity strata. The wide moderate-stratum confidence interval reflects near-complete outcome separation in the hyperglycemic group (11 of 12 unfavorable vs 3 of 11 in the euglycemic stratum). Right-pointing arrow indicates the upper confidence limit for the moderate stratum extends beyond the displayed axis. CI = confidence interval; mRS = modified Rankin Scale; NIHSS = National Institutes of Health Stroke Scale; OR = odds ratio.

**Supplementary Figure S3. Heatmap of inappropriate glycemic management prevalence by NIHSS group at presentation and admission hyperglycemia status.**

Each cell shows the percentage of patients with inappropriate glycemic management within that stratum, with the underlying fraction (patients with inappropriate management over total patients in the stratum). The pattern indicates that admission hyperglycemia, not stroke severity, is the principal driver of management failure across all NIHSS strata. NIHSS = National Institutes of Health Stroke Scale.

**Supplementary Figure S4. Unfavorable discharge mRS rate across stress hyperglycemia ratio (SHR) tertiles.**

Bar chart of unfavorable discharge mRS rate (per cent with mRS 3–6) across SHR tertiles (n = 165). Tertile boundaries: T1 ≤0.873, T2 0.873–1.059, T3 >1.059. SHR was calculated using the Roberts formula. Error bars show 95 per cent Wilson confidence intervals for proportions. Linear trend across tertiles: p = 0.002 (odds ratio per tertile = 1.90, unadjusted). After full nine-covariate adjustment (Table 6), the tertile 3 versus tertile 1 odds ratio was 2.45 (95 per cent CI 0.90–6.66, p = 0.078). CI = confidence interval; mRS = modified Rankin Scale; SHR = stress hyperglycemia ratio.

***Supplementary Figure S1. Receiver operating characteristic (ROC) curve for the primary multivariable model of unfavorable discharge mRS.***

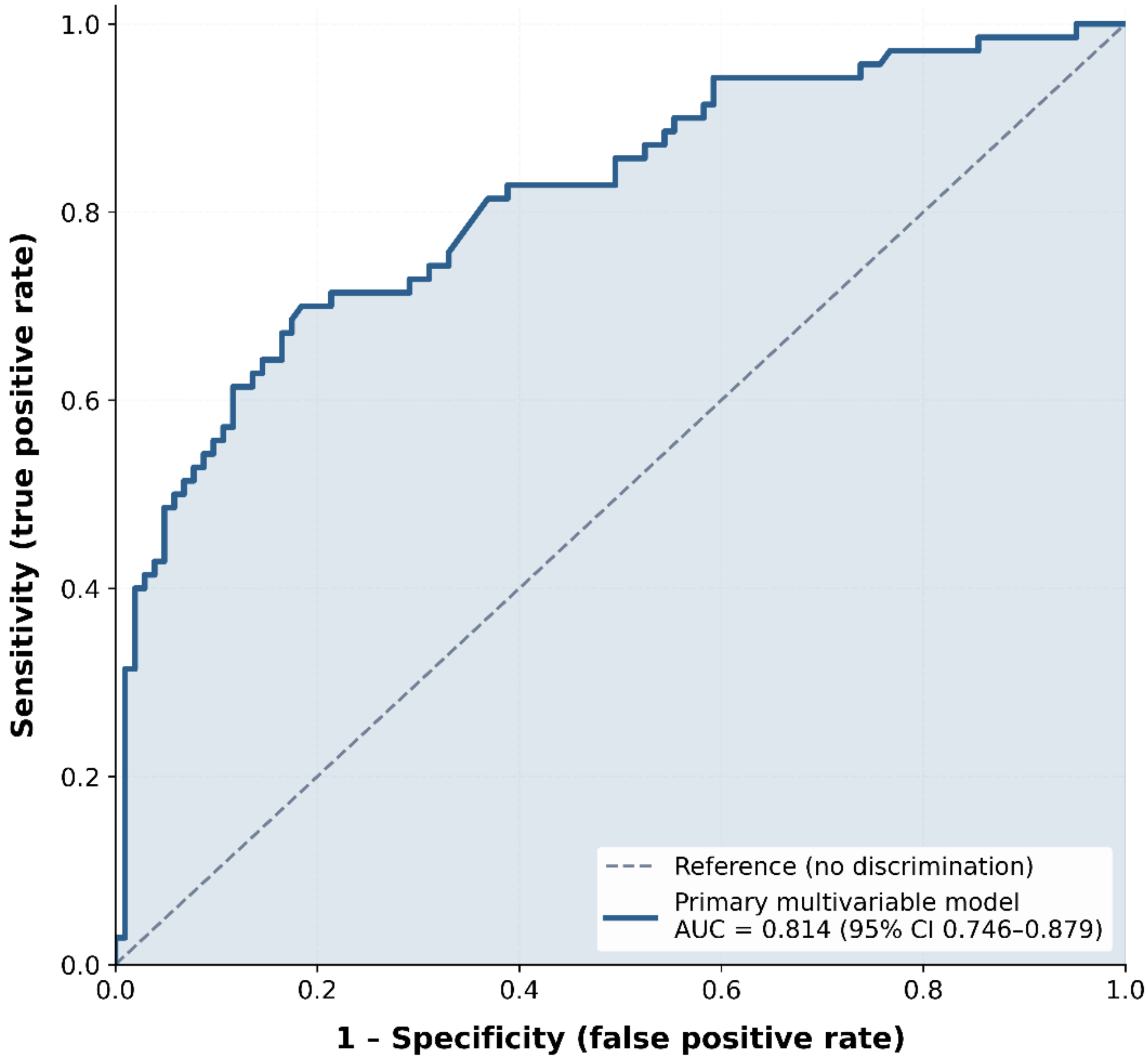

Sensitivity (true positive rate)
1.0
0.8
0.6
0.4
0.2
0.0
0.0
0.2
0.4
0.6
0.8
1.0
1 – Specificity (false positive rate)
Reference (no discrimination)
Primary multivariable model
AUC = 0.814 (95% CI 0.746–0.879)

***Supplementary Figure S2. Stratified analysis of admission hyperglycemia by NIHSS group.***

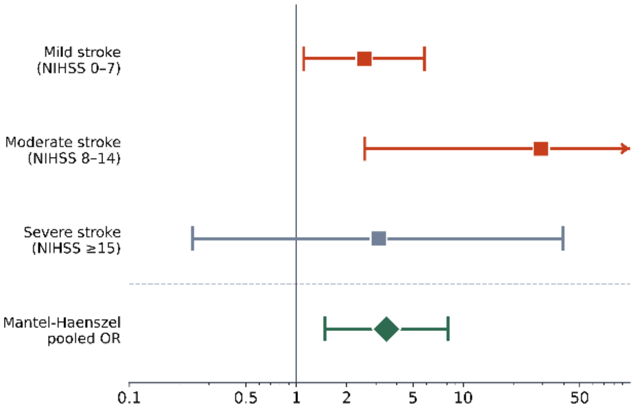


| | Hyperglycaemic events / N (%) | Euglycaemic events / N (%) | OR (95% CI) |
|---|---|---|---|
| Mild stroke (NIHSS 0–7) | 22/62 (35.5%) | 11/62 (17.7%) | 2.55 (1.11–5.87) |
| Moderate stroke (NIHSS 8–14) | 11/12 (91.7%) | 3/11 (27.3%) | 29.33 (2.56–336.4) |
| Severe stroke (NIHSS ≥15) | 14/15 (93.3%) | 9/11 (81.8%) | 3.11 (0.24–39.54) |
| Mantel-Haenszel pooled OR | *n = 173 (pooled)* | | **3.47 (1.48–8.10)** |

***Supplementary Figure S3. Heatmap of inappropriate glycemic management prevalence by NIHSS group at presentation and admission hyperglycemia status.***

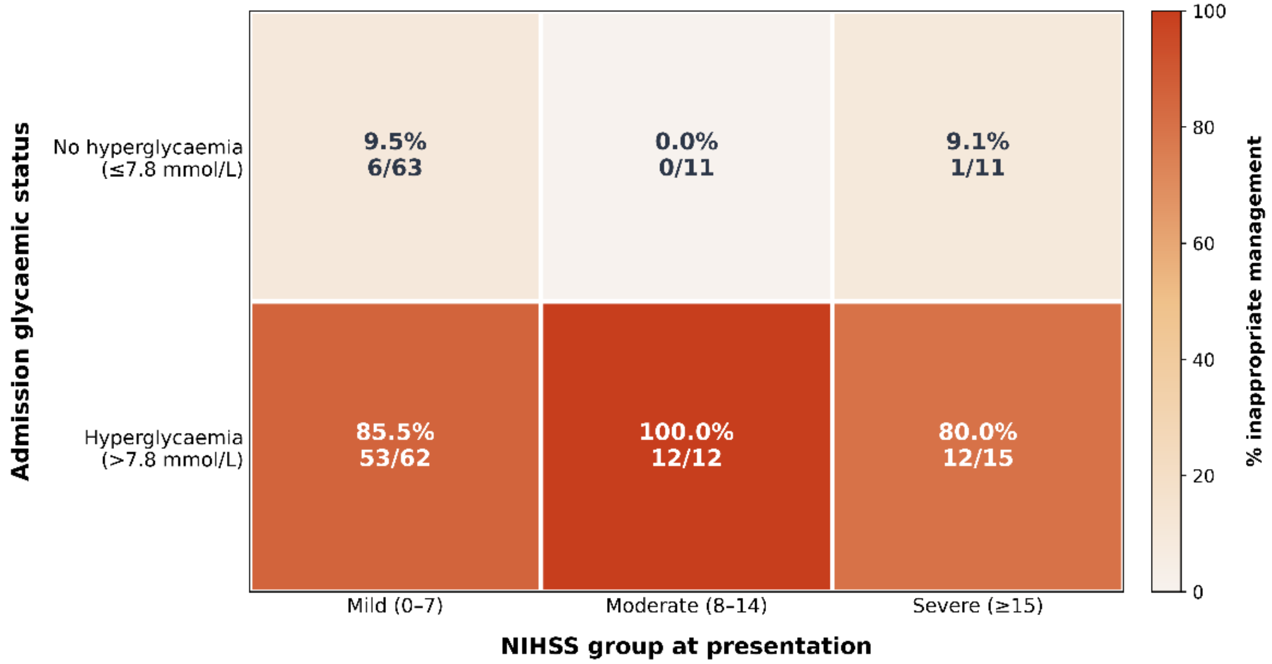

*Supplementary Figure S4. Unfavorable discharge mRS rate across stress hyperglycemia ratio (SHR) tertiles.*

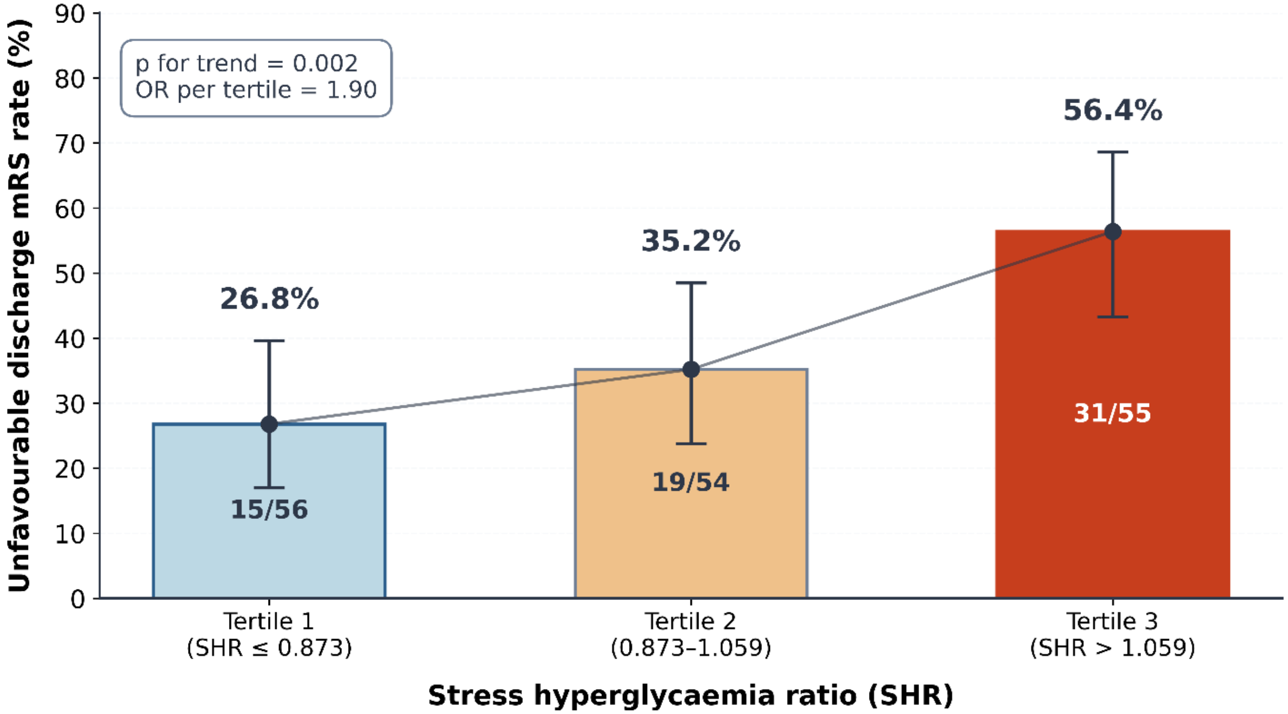

Supplement: Supplementary file 1 [file SupplementaryFile1.pdf]
